# A novel *MMP13* frameshift variant causes short stature via enhanced MMP13–HSPA5 interaction and activated endoplasmic reticulum stress

**DOI:** 10.1002/ctm2.70648

**Published:** 2026-03-24

**Authors:** Huifei Lu, Xin Feng, Suping Dai, Yilin Zhu, Ke Yuan, Yonghua Chen, Jianfang Zhu, Yanlan Fang, Qingfeng Yan, Chunlin Wang

**Affiliations:** ^1^ Department of Pediatrics, The First Affiliated Hospital Zhejiang University School of Medicine Hangzhou China; ^2^ College of Life Science Zhejiang University Hangzhou China

**Keywords:** endoplasmic reticulum stress, extracellular matrix, growth plate, HSPA5, MMP13, short stature, unfolded protein response

## Abstract

**Background:**

Short stature (SS) is a common growth disorder with multiple aetiologies. Variants in the *MMP13* gene can result in varying degrees of SS, typically accompanied by pronounced skeletal abnormalities. This study aimed to investigate the genetic basis of SS in a family lacking significant imaging abnormalities and elucidate the underlying pathogenic mechanism.

**Methods:**

Trio whole‐exome sequencing was performed in a Chinese pedigree with SS to identify pathogenic variants, followed by Sanger sequencing validation. Patient‐derived induced pluripotent stem cell model and CRISPR/Cas9‐generated Mmp13^R459fs^ homologous mutant mouse model were established to verify the pathogenicity of the variant. Western blotting, immunofluorescence staining, co‐immunoprecipitation coupled with mass spectrometry (Co‐IP/MS), histological staining and transmission electron microscopy were used to evaluate the effects of the variant on MMP13 protein function and chondrocyte development.

**Results:**

A heterozygous frameshift variant, NM_002427.4:c.1372del(p.Arg458Valfs*31), was identified in the *MMP13* gene. Mmp13^R459fs^ mutant mice recapitulated the SS phenotype in patients, with growth plate abnormalities that were present only during the growth phase and resolved earlier than those in *Mmp13* knockout mice. Co‐IP/MS in HEK293T cells revealed significantly increased HSPA5 expression in the mutant, and enhanced interaction between MMP13 mutant and HSPA5 was confirmed, leading to their retention within the endoplasmic reticulum (ER). In patient‐derived chondrocytes, misfolded MMP13 protein upregulated HSPA5 expression, induced significant ER dilation, activated unfolded protein response and increased chondrocyte apoptosis, ultimately contributing to *MMP13*‐related SS.

**Conclusion:**

This study for the first time reports the *MMP13* c.1372del (p.Arg458Valfs*31) variant causes autosomal dominant SS without obvious skeletal abnormalities. The variant is associated with defective MMP13 protein secretion and ER stress. These findings expand the mutational spectrum and genotype‒phenotype correlations of the *MMP13* gene, providing a novel pathogenic mechanism of SS that is important for the precise diagnosis and treatment.

**Key points:**

- The MMP13 R458fs variant is retained in the endoplasmic reticulum (ER), leading to ER expansion.
- Enhanced binding of variant MMP13 to HSPA5 triggers ER stress, thereby increasing chondrocyte apoptosis.
- This pathogenic cascade results in abnormal expansion of the growth plate hypertrophic zone, ultimately impairing long bone growth and causing short stature.

## Graphical abstract

The MMP13 R458fs protein is retained in the ER and exhibits enhanced binding to HSPA5, which promotes the dissociation of IRE1α, ATF6, and PERK. This triggers ER stress, leading to increased chondrocyte apoptosis and abnormal expansion of the growth plate hypertrophic zone, ultimately impairing long bone growth and causing short stature.

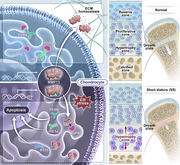

## 1 INTRODUCTION

Human height is an inheritable polygenic trait regulated by multiple complex genes.^1^, ^2^ Short stature (SS) is defined as a body height of more than 2 standard deviations (SDs) below the population height standard for a given age, sex and demographic environment.^3^ SS is among the most common endocrine‐associated phenotypic manifestations in childhood, presenting either as an isolated finding or in association with other clinical phenotypes, such as skeletal dysplasia and growth retardation.^4^ SS can result from various mechanisms, including primary disorders of growth plate and long bones, growth hormone (GH) deficiency or insensitivity, and disruptions of fundamental cellular processes, extracellular matrix (ECM), paracrine signalling pathways or hormonal signalling pathways.^2^

Genetic factors are closely related to the development of human height. The development of comprehensive sequencing technologies, particularly next‐generation sequencing, has facilitated the identification of genetic defects underlying monogenic growth disorders. An increasing number of genes associated with SS have been identified, many of which are involved in key physiological processes regulating growth plate function and long bone development. These genes participate in hormonal regulation, paracrine signalling pathways and composition of ECM components. They also support essential intracellular processes such as chondrocyte proliferation, hypertrophy and ECM production, which are fundamental to skeletal growth. Variants in genes encoding cartilage ECM proteins (e.g., *COL2A1*, *MATN3*, *COMP*, *ACAN*, *FBN1* and *MMP13*) can disrupt growth plate chondrogenesis, leading to the growth failure with a broad phenotypic spectrum.^5^, ^6^, ^7^, ^8^ These variants may also affect connective tissues beyond the growth plate, resulting in various skeletal and joint problems and, occasionally, medical problems involving other organ systems.^1^

*MMP13* gene variants are associated with SS, which can be classified into two distinct categories based on their inheritance patterns. Spondyloepimetaphyseal dysplasia, Missouri type (SEMD_MO_) or metaphyseal anadysplasia 1 (MANDP1) [MIM#602111] is an autosomal dominant genetic disorder, whereas metaphyseal dysplasia, Spahr type [MIM#250400] is an autosomal recessive genetic disorder.^9^, ^10^, ^11^ It has been reported that SS related to *MMP13* variants is often accompanied by skeletal abnormalities (Table 1). As shown in Table 1, SS related to *MMP13* variants has a broad spectrum of clinical manifestations. Notably, variations in height were observed even among individuals within the same family carrying an identical mutation. In autosomal dominant families, all but one (for whom clinical details were not specified) presented with gait disturbance or genu varum, and all the patients with available radiographic data exhibited skeletal abnormalities. In this study, a family with SS was examined, and trio whole‐exome sequencing (Trio‐WES) identified a heterozygous frameshift variant in the *MMP13* gene. However, no obvious abnormalities were found on X‐ray imaging.

**TABLE 1:** Summary of previously reported *MMP13* variants.

Description	Inheritance/mutation	Sex	Age	Height	Clinical features	Radiology
Diaz Escagedo et al. (2021)	AD/p.Gln72His	Male	1Y6M	1st centile	Waddling gait	Broad and irregular humerus, femoral neck, distal femur, proximal tibia and proximal fibula
		Male	n.d.	165 cm	n.d.	Occult spina bifida
		Female	n.d.	<150 cm	n.d.	n.d.
Kennedy et al. (2005)	AD/p.Phe75Ser	Male	n.d.	n.d.	Bowed legs, coxa vara	Pear‐shaped vertebrae, metaphyseal irregularities
Lausch et al. (2009)	AD/p.Phe74Ser	Female	6Y	‒2.35SD	Severe bowed legs in infancy	Irregular metaphyses of the ulnae, radii, tibiae and femora; normal spine
		Female	39Y	‒1.72SD	Severe bowed legs in infancy	n.d.
		Female	65Y	‒2.58SD	Severe bowed legs in infancy and persisted	n.d.
Lausch et al. (2009)	AD/p.Met91Thr	Male	18Y	+1.6SD	Bowed legs in infancy	Metaphyseal irregularities
		Male	15Y	‒.89SD	Bowed legs in infancy	n.d.
		Male	42Y	+1.2SD	Bowed legs in infancy	n.d.
		Male	62Y	+.72SD	Bowed legs in infancy	n.d.
Lausch et al. (2009)	AD/p.Met91Thr	Female	6Y	‒2.89SD	Bowed legs in infancy	n.d.
		Female	3Y1M	‒1.54SD	Bowed legs in infancy	n.d.
		Female	32Y	‒1.9SD	Bowed legs in infancy	n.d.
Song et al. (2019)	AR/p.Met71Thr	Fe	3Y9M	‒2SD	Waddling gait; walked independently at 1.5 years	Metaphyseal irregularities, pear‐shaped vertebrae, delayed epiphyseal ossification
Li et al. (2015)	AR/p.Arg109Ter	Male	14Y9M	149.8 cm; ‒2.6SD	Chronic knee pain	Lower limb metaphyseal irregularities, unremarkable upper limbs and spine
		Male	7Y5M	114 cm; ‒1.9SD	Waddling gait; bowed legs, coxa vara, pes planus	Distal femoral metaphyseal irregularity, normal spine
Bonafé et al. (2014)	AR/p.Trp207Gly	Female	47Y	138 cm	Chronic knee pain	MR: osteoarthritis and osteochondritis dissecans of the knee
		Male	n.d.	167 cm	Healthy	n.d.
		Male	n.d.	150 cm	Healthy	n.d.
		Female	31Y	150 cm	Knee pain in childhood, asymptomatic in adulthood	n.d.
Tadros et al. (2017)	AR/p.Trp207Gly	Female	11Y	.4^th^ centile	Bowed legs	Tibial and ulnar metaphyseal irregularities, normal bone age
		Male	6Y	9^th^ centile	Mild bowed legs	Irregular metaphyses, most marked in the distal tibia, normal bone age
Lausch et al. (2009)	AR/p.His213Asn	Male	3Y2M	‒1.0SD	Bowed legs in infancy	n.d.

Abbreviations: AD, autosomal dominant; AR, autosomal recessive; M, month; MR, magnetic resonance; n.d., not determined; SD, standard deviation; Y, year.

MMP13 belongs to the matrix metalloproteinase (MMP) family. *MMP13* gene (MIM*600108) maps to 11q22.2, spans 2714 bp in length, consists of 10 exons and contains a 1416‐bp protein‐coding sequence.^12^ It encodes MMP13 protein (also known as collagenase 3), which is primarily localised in the ECM.^13^, ^14^ MMP13 protein encompasses three principal domains: an N‐terminal pro‐peptide domain, a catalytic domain and a C‐terminal hemopexin‐like domain, with the latter two connected by a flexible hinge region.^15^ In the pro‐peptide domain, a cysteine residue coordinates with the catalytic zinc within the active site, thereby blocking substrate access and maintaining MMP13 in its latent zymogen form.^16^ The catalytic domain of MMP13 is located in the central portion of the protein, which harbours all calcium‐ and zinc‐binding residues essential for enzymatic activity, specifically within amino acid residues 172‒232.^11^ As a protease critical for skeletal development, MMP13 degrades diverse ECM components, including fibrillar collagen, fibronectin and aggrecan. Exclusively expressed in the cartilage growth plate and primary ossification centres during embryogenesis, it is indispensable for both endochondral and intramembranous ossification, thereby playing a pivotal role in embryonic bone development and subsequent postnatal remodelling.^17^, ^18^ MMP13 is synthesised by terminally differentiated hypertrophic chondrocytes and osteoblasts.^14^, ^15^, ^19^ MMP13 deficiency may disrupt the degradation of ECM in the growth plate, leading to the accumulation of type II and type X collagens, delayed endochondral ossification and impaired biological processes (e.g., chondrocyte differentiation, apoptosis and matrix remodelling).^14^ Abnormal MMP13 expression or MMP13 dysfunction is associated with various skeletal disorders, including osteoarthritis,^20^ chondrodysplasia and bone tumours.^21^ *Mmp13* knockout mice present with distinct skeletal phenotypes, including an expanded growth plate hypertrophic zone, increased trabecular bone density and delayed longitudinal bone formation. These phenotypes result from disrupted ECM remodelling, which prolongs chondrocyte survival, delays vascular invasion,^15^ and leads to defective trabecular bone formation.^22^ Notably, however, skeletal alterations in *Mmp13* knockout mice are evident during the growth phase, with the most severe growth plate abnormality observed at 5 weeks of age. As longitudinal bone growth slows, hypertrophied chondrocytes gradually return to normal. By 12 weeks of age, when longitudinal bone growth has ceased entirely, the growth plate abnormality resolves.^15^, ^22^

Kennedy et al. for the first time proposed that SEMD_MO_ was related to heterozygous *MMP13* variants.^23^ They reported that the MMP13 F75S variant induced intracellular misfolding of the mutant protein, leading to autoactivation and autodegradation.^23^ This process culminates in the release of small, enzymatically inactive fragments, ultimately contributing to the presence of SEMD_MO_. Despite these findings, the precise mechanisms by which *MMP13* variants cause SEMD remain unclear, and no effective treatments are currently available. Therefore, clarifying the pathogenicity of *MMP13* variants and their regulatory mechanisms in skeletal development is therefore crucial for establishing a theoretical basis for future precise diagnosis and therapy of SS.

In the present study, an SS pedigree was found to harbour an *MMP13* variant without obvious skeletal abnormalities on X‐ray imaging. To further confirm the pathogenicity of this *MMP13* variant, a patient‐specific induced pluripotent stem cell (iPSC) model and a mouse model carrying the homozygous variant were established. Our study for the first time reported that *MMP13* variant upregulated HSPA5 and activated endoplasmic reticulum (ER) stress, revealing a potential pathogenic mechanism by which *MMP13* variant contributes to SS. Our results provide novel insights for the precise diagnosis and therapy of SS.

## 2 METHODS

### 2.1 Study approval

This study was approved by the Ethics Committee of The First Affiliated Hospital of Zhejiang University School of Medicine (no. 1468 in 2024) and conducted in full accordance with the Declaration of Helsinki. Written informed consent was obtained from the proband's parents prior to the study.

### 2.2 Clinical evaluations

The proband and her parents underwent detailed assessment of medical history and clinical symptom. The proband was a 6‐year‐and‐3‐month‐old girl who presented to our clinic with growth retardation for 4 years. Her annual growth rate was approximately 5 cm, and her growth curve remained in the third centile. Physical examination indicated a height of 107.6 cm (‒2.2 SD), an upper body segment of 55.3 cm, a lower body segment of 52.3 cm (upper‐to‐lower segment ratio of 1.06) and a weight of 18.1 kg. She exhibited normal cognitive development and nutritional status without distinctive facial features. She was born at full term, measuring 50 cm in length and weighing 3200 g. She was born to non‐consanguineous parents. Both parents were physically healthy. The height of her father and mother was 150.8 cm (‒3.59 SD) and 153.9 cm (‒1.24 SD), respectively. The musculoskeletal assessment of joints and skeletal system was performed, including evaluations of joint mobility, spinal alignment and limb proportions. Subsequent imaging examinations included bilateral full‐length lower limb radiography and anteroposterior pelvic radiography. A comprehensive laboratory evaluation was conducted for the proband, including measurements of blood insulin‐like growth factor‐1 (IGF‐1), liver and kidney function tests, detection of blood electrolytes, thyroid function test, karyotyping and bone age assessment via radiography.

### 2.3 Whole‐exome sequencing

Genomic DNA was isolated from peripheral blood leukocytes of the proband and her parents using standard protocols, and Trio‐WES was performed. The identified *MMP13* variant was confirmed by Sanger sequencing and annotated based on NCBI reference NG_021404.1 (NM_002427.4). All variant designations conform to the Human Genome Variation Society nomenclature guidelines.

### 2.4 Molecular modelling

To assess the structural impact of the identified *MMP13* variant, the 3D conformation of human MMP13 predicted by AlphaFold (ID: AF‐P45452‐F1) was analysed. Molecular graphics and structural visualisations were generated using PyMOL 2 software.

### 2.5 PCR and Sanger sequencing

PCR amplification of genomic fragments encompassing the variant detected by Trio‐WES was performed, followed by bidirectional Sanger sequencing using the primers listed in Table S3.

### 2.6 Generation of iPSCs

iPSCs were established by nucleofection with episomal plasmids harbouring reprogramming factors OCT4, SOX2, KIF4, c‐MYC and LIN28.^24^ The pluripotency of iPSCs was validated by assessment of pluripotent gene expression, karyotype analysis and alkaline phosphatase staining. Mycoplasma contamination was systematically screened in all iPSCs. A comprehensive description of the protocols^25^ is available in the Supporting Information.

### 2.7 Cell culture

iPSCs were cultured in mTeSR1 medium (STEMCELL Technologies) on Matrigel‐coated plates (Corning). HEK293T cells were maintained in high‐glucose Dulbecco's Modified Eagle Medium (DMEM; Invitrogen) supplemented with 10% foetal bovine serum (FBS). Both cell types were grown at 37°C under a 5% CO_2_ atmosphere.

### 2.8 Mesenchymal stem cell and chondrogenic differentiation

iPSCs were induced to differentiate into mesenchymal stem cells (MSCs) with the Stemdiff Mesenchymal Progenitor Kit (STEMCELL Technologies) in accordance with the manufacturer's instructions. Subsequently, cells were cultured in the MesenCult‐ACF Chondrogenic Differentiation medium (STEMCELL Technologies) for 6 weeks (Figure S1C). The medium was refreshed every other day.

### 2.9 Flow cytometry

For immunophenotyping of MSCs, the following mouse anti‐human monoclonal antibodies were employed: CD73‐FITC (Beyotime, AC0346), CD90‐FITC (Beyotime, AC0355), CD34‐PE (Beyotime, AC0535) and CD45‐PE (Beyotime, AC0548). Prior to staining, 2.5‒5.0 × 10^5^ cells were harvested and washed with flow cytometry staining buffer according to the manufacturer's instructions. Then, cells were incubated with 2.5 µL of antibody for 30 min at 4°C in dark, followed by flow cytometry.

### 2.10 Establishment of Mmp13^R459fs^ mouse model

Using the CRISPR/Cas9 system, a mouse model carrying the Mmp13^R459fs^ variant (homologous to human MMP13^R458fs^) was established via fertilised egg microinjection. A gRNA (CACCACAATATGGAATTTGT) was designed to direct Cas9‐mediated cleavage, and a homologous recombination vector was constructed for precise editing. Following microinjection of Cas9, gRNA and the donor vector into fertilised eggs of C57BL/6J mice, viable embryos were transplanted into pseudo‐pregnant female mice. Genotyping of F0 progeny was performed by PCR and sequencing, confirming successful germline transmission. All animal experiments were conducted with approval from the Zhejiang University Animal Ethics Committee.

### 2.11 Body length and femur/tibia measurements

Mice were anaesthetised with isoflurane. Once anaesthesia was confirmed by the absence of toe‐pinch reflex, body length was measured as the distance from the nose tip to the anal base with the animal in a relaxed supine position. Triplicate measurements per mouse were averaged. Twelve‐week‐old mice were anaesthetised with isoflurane before X‐ray radiography. ImageJ software, with the measurement landmarks defined as follows: the proximal end of the femur at the top of the femoral head and the distal end at the lowest point of the intercondylar fossa; the proximal end of the tibia at the highest point of the tibial plateau and the distal end at the lowest point of the medial malleolus. Each bone length was measured in triplicate; the average of three valid measurements was recorded as the final result.

### 2.12 Safranin O/Fast Green, Alcian blue and haematoxylin‒eosin staining

Mice were euthanised, and the hindlimbs were harvested, immersed in 4% paraformaldehyde solution at room temperature for 48 h, and subsequently decalcified in ethylenediaminetetraacetic acid (EDTA, pH 7.2). After 20 days of decalcification, specimens were embedded in paraffin and sectioned at a thickness of 5 µm. The sections were rehydrated and stained with Safranin O/Fast Green, Alcian and haematoxylin‒eosin (HE) for histological examination of growth plate structure. Given the growth plate's uneven thickness, 10 evenly spaced measurements were taken across its width to assess the total thickness and thickness of proliferative and hypertrophic zones. The average of 10 measurements was used as the representative thickness for the growth plate or respective zones.

### 2.13 Plasmid construction and cell transfection

Wild‐type (WT) human MMP13 cDNA (NM_002427.4) was synthesised and cloned into a p3×Flag‐CMV‐14 vector. Mutant constructs (c.224T>C and c.1372del) were generated using the In‐Fusion HD Cloning Kit (TaKaRa), and additional plasmids for co‐immunoprecipitation (Co‐IP) (HSPA5‐3×HA, MMP13 ΔPro‐3×Flag, MMP13 ΔCat‐3×Flag and MMP13 ΔHex‐3×Flag) were constructed. All plasmids were transformed into *Escherichia coli* DH5α, and the insert integrity was confirmed by sequencing (primers listed in Table S4). HEK293T cells were transfected with the validated constructs using Liposomal 2000 Transfection Reagent (Yeasen, 40802ES08). The culture medium was replaced with DMEM supplemented with 10% FBS after 6–8 h. Cells were harvested after 48 h.

### 2.14 Western blotting

Cells were harvested and lysed in buffer (50 mM Tris‒HCl, 150 mM NaCl, 1 mM EDTA,  .5% NP40) supplemented with protease inhibitors, and centrifuged at 12 000 × g for 10 min at 4°C to obtain the supernatant. Protein concentrations were measured using the BCA Protein Assay Kit (Beyotime, P0009). Equal amounts of protein (20 µg per lane) were separated by 10% SDS‒PAGE and transferred to polyvinylidene fluoride (PVDF) membranes for immunoblotting. The manufacturers and catalogue numbers of antibodies used in this study are provided in Table S5, and all experiments were conducted with at least three independent replicates.

### 2.15 Antibodies

Specific antibodies were purchased for indicated experiments: anti‐MMP13 (69926, 1:1000 for Western blotting [WB]), anti‐SOX‐2 (3579, 1:50 for immunofluorescence [IF]), anti‐OCT‐4 (2750, 1:50 for IF), anti‐NANOG (4903, 1:50 for IF), anti‐GAPDH (2118, 1:10 000 for WB), anti‐FLAG (14793, 1:1000 for WB, 1:50 for IF), anti‐HSPA5 (3177, 1:1000 for WB), anti‐SSEA4(4755, 1:50 for IF), anti‐TRA‐1‐60 (4746, 1:50 for IF), anti‐GRP94 (2104, 1:1000 for WB), anti‐PERK (3292, 1:1000 for WB), anti‐IRE1α (3294, 1:1000 for WB) and anti‐ATF6 (65880, 1:1000 for WB) from CST; anti‐MMP13 (ab39012, 1:2000 for WB), anti‐IRE1α (ab37073, 1:50 for IF), anti‐PERK (ab217322, 1:50 for IF) and anti‐ATF6 (ab37149, 1:50 for IF) from Abcam; anti‐MMP13 (18165‐1‐AP, 1:10 000 for WB), anti‐ATF4 (10835‐1‐AP, 1:10 000 for WB) and anti‐CHOP (15204‐1‐AP, 1:10 000 for WB) from Proteintech; anti‐KLF4 (C3252, 1:50 for IF) from Beyotime.

### 2.16 RNA extraction and quantitative PCR

Total RNA was extracted with TRIzol (Invitrogen) and reverse‐transcribed into cDNA using the PrimeScript RT Reagent Kit with gDNA Eraser (Takara). Quantitative real‐time PCR (qPCR) was performed on an ABI PRISM 7900HT System (Applied Biosystems) with primers listed in Table S6. GAPDH was used as an internal control for normalisation.

### 2.17 Immunofluorescence staining

HEK293T cells and iPSCs grown on glass coverslips were fixed with 4% paraformaldehyde (30 min), permeabilised with  .2% Triton X‐100 (30 min) and blocked with 3% bovine serum albumin (BSA) (2 h) at room temperature. These cells were then incubated with primary antibodies (4°C, overnight) in 1% BSA, followed by incubation with corresponding secondary antibody, and counterstained with 4′,6‐diamidino‐2‐phenylindole (DAPI) (10 ng/mL) for 10 min at room temperature.

### 2.18 Protein purification

HEK293T cells were transfected with Flag‐HA‐HSPA5, Flag‐MMP13 WT or Flag‐MMP13 R458fs plasmids using Hieff Trans Liposomal 2000 (Yeasen, 40802ES08). After 48 h, cells were lysed in non‐denaturing buffer; protein complexes were isolated with FLAG/HA beads, washed with pull‐down buffer (50 mM Tris‒HCl, 300 mM NaCl, 1 mM EDTA,  .5% NP40), and analysed by 10% SDS‒PAGE followed by Coomassie Brilliant Blue staining to assess purity.

### 2.19 Co‐immunoprecipitation assay and mass spectrometry

Flag‐tagged proteins were incubated with Flag affinity beads (Sigma‒Aldrich) overnight at 4°C, followed by three washes with lysis buffer (3 min each). Bound proteins were eluted by boiling in 2× SDS loading buffer (50 µL) for 10 min, and then analysed by SDS‒PAGE and mass spectrometry (MS).

### 2.20 Enrichment of ER

The ER Enrichment Kit (Invent Biotechnologies) was employed to isolate ER according to the manufacturer's instructions. The detailed procedures for enrichment of ER are available in the Supporting Information.

### 2.21 Transmission electron microscopy

Chondrogenic samples were fixed overnight in 2.5% glutaraldehyde at 4°C. Ultrathin sections were then examined using a Tecnai G2 Spirit transmission electron microscope operating at 120 kV at the Center of Cryo‐Electron Microscopy, Zhejiang University.

### 2.22 TUNEL staining

TUNEL assays were performed on paraffin‐embedded iPSC‐derived chondrogenic samples from 42 days and hindlimb sections from 4‐week‐old mice according to the manufacturer's instructions. Following fluorescent labelling of fragmented DNA, sections were counterstained with DAPI, and TUNEL‐positive cells were quantified using ImageJ software.

### 2.23 Statistical analysis

All data, unless otherwise specified, were obtained from a minimum of three independent experiments. Statistical differences between groups were evaluated using an unpaired, two‐tailed Student's *t*‐test. A value of *p* < .05 was considered statistically significant. Data are expressed as mean ± standard error (*n* ≥ 3).

## 3 RESULTS

### 3.1 Clinical characterisation of the patient and molecular genetic diagnosis

**FIGURE 1:**
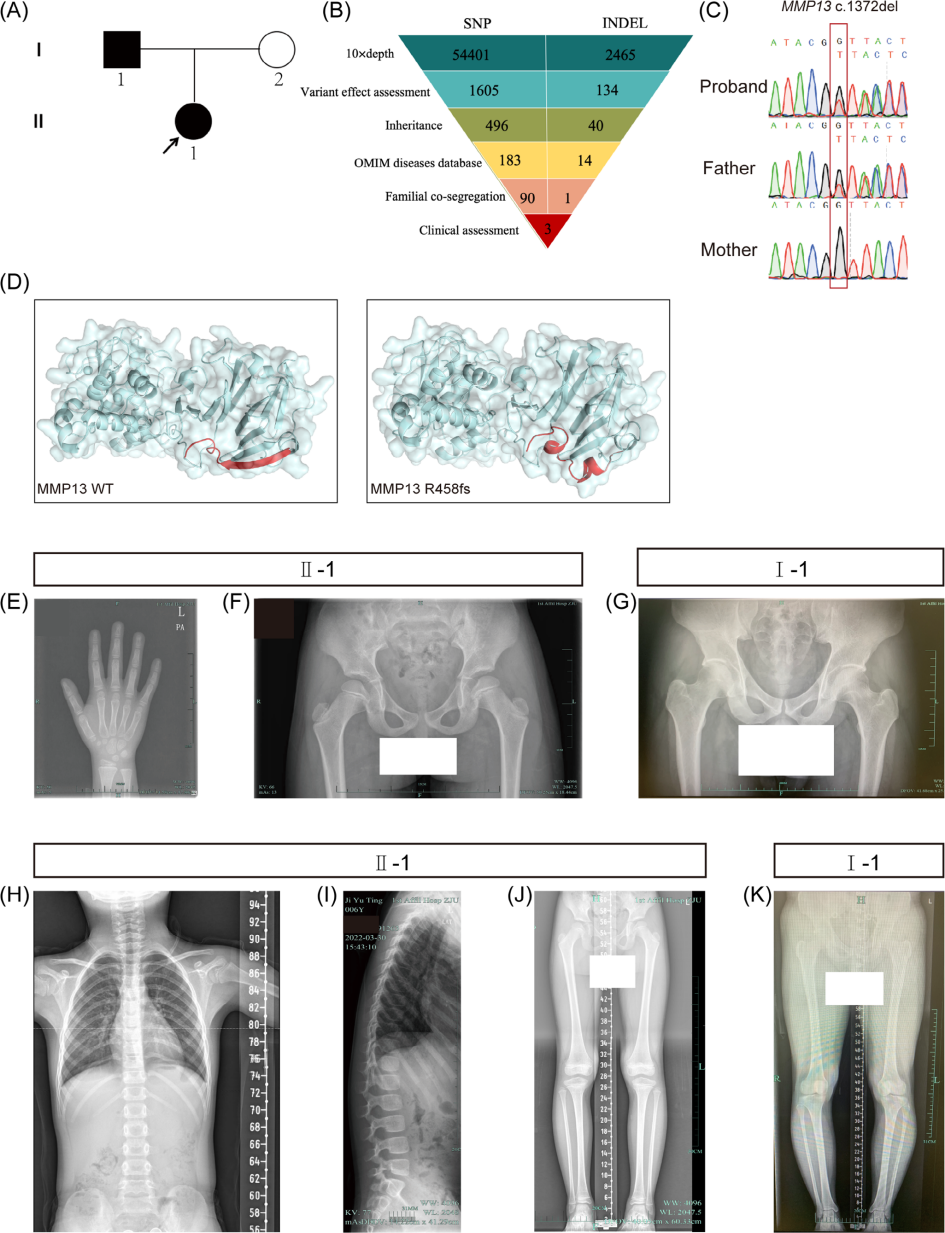
Genetic testing and radiographical features of a Chinese family with short stature. (A) Pedigree of short stature (SS) family. Squares and circles indicate males and females, respectively. The proband is indicated by arrow. Affected individuals are represented by filled black symbols, while unaffected individual (harbouring the wild‐type sequence) is denoted by open symbol. (B) Schematic representation of exome‐data‐filtering approach, which assumed a dominant inheritance pattern in the family. (C) Sanger sequencing chromatograms confirmed the proband and her father carried the c.1372del heterozygous frameshift variant in the *MMP13* gene. (D) Three‐dimensional structure analysis of MMP13. The red fragment represents the amino acids from the variant site to the stop codon. MMP13 R458fs variant resulted in an extension of the encoded amino acids. Radiographic of subjects with SS. (E) The bone age was 5 years at the actual age of 6.25 years. (F and G) X‐ray images of the bilateral hips of the proband and her father showed normal development of both femoral heads, with no evident bilateral coxa vara. (H and I) Anteroposterior and lateral radiographs of the proband's spine. The physiological curvature of spine was observed without lordosis or pear‐shaped changes. (J and K) Full‐length X‐ray images of both lower extremities in the proband and her father showed no widening of the metaphyses in the bilateral femurs, tibias and fibulas, with regular margins.

The proband was a 6‐year‐and‐3‐month‐old girl with a height of 107.6 cm (‒2.2 SD). The physical examination indicated symmetrical SS (an upper‐to‐lower segment ratio of 1.06), within the normal range. Her annual height growth rate was approximately 5 cm, and serum IGF‐1 level was normal, ruling out growth hormone deficiency. Laboratory analysis of blood samples indicated normal liver, kidney and thyroid functions. Serum calcium, phosphorus and alkaline phosphatase levels were also within normal limits. Chromosome karyotyping demonstrated a 46, XX pattern (Table S2). Therefore, hypothyroidism and Turner syndrome were excluded.^26^ Bone age analysis after left hand‐wrist radiography showed 5 years (according to the Greulich‒Pyle standards), representing a delay of more than 1 year, with no evidence of metaphyseal dysplasia (Figure 1E).

A comprehensive pedigree investigation was conducted in this pedigree. The proband's father exhibited significant SS (Figure 1A) and experienced knee discomfort during adolescence but was asymptomatic in adulthood. No additional clinically significant symptoms or signs were observed in other family members.

### 3.2 Genetic diagnoses

The core pedigree members underwent Trio‐WES and validation was done by Sanger sequencing. Initial analysis identified 56 866 variants within the exome region. Following sequential filtering, 197 variants remained, which were further narrowed to three rare variants that exhibited familial co‐segregation and were associated with SS or skeletal dysplasia (Table S1). Among these, *ARCN1* gene variants have been reported to be associated with SS‐micrognathia syndrome, characterised by rhizomelic SS accompanied by microcephaly, micrognathia and growth retardation.^27^ However, the proband and his father did not present with these phenotypes, and ClinVar classified this variant as benign. The *FGFR1* gene variant was synonymous and designated as likely benign in ClinVar. Ultimately, a frameshift variant in the *MMP13* gene (NM_002427.4) was identified in the proband (Figure 1B). Sanger sequencing confirmed the presence of a heterozygous c.1372del (p. Arg458Valfs*31) variant. This variant has been registered in population databases (rs782085134) and is listed in ClinVar (Variation ID: 631644). However, it has never been reported in individuals with MMP13‐related conditions. The variant changed the encoded arginine to valine and introduced a termination codon 30 amino acids downstream, ultimately resulting in an 18‐amino acid extension of the protein (Figure 1D). Although it was not anticipated to result in nonsense‐mediated decay, this frameshift variant was predicted to disrupt the C‐terminal residues of MMP13 protein. It was present at a low frequency in population databases; specifically, it is reported in gnomAD (.01% in exomes), but not recorded in 1000 Genomes or ESP6500. Collectively, the available data were currently inadequate to assign a definitive pathogenic role of this variant. According to the ACMG/AMP variant interpretation standards, it was classified as a variant of uncertain clinical significance. Subsequent Sanger sequencing of parental samples indicated that the proband's father carried the same variant and exhibited SS phenotypes, consistent with genetic cosegregation (Figure 1C).

The proband underwent comprehensive imaging evaluations, including anteroposterior and lateral spinal radiography, bilateral lower limb radiography and anteroposterior pelvic radiography (Figure 1F,H,I,J). Additionally, the proband's father also underwent radiographic assessments, including anteroposterior pelvic radiography and bilateral full‐length lower‐limb radiography (Figure 1G,K). However, no significant abnormal radiographic findings were observed in either affected individual.

### 3.3 Generation of SS patient‐iPSCs and their differentiation into chondrogenic pellets

**FIGURE 2:**
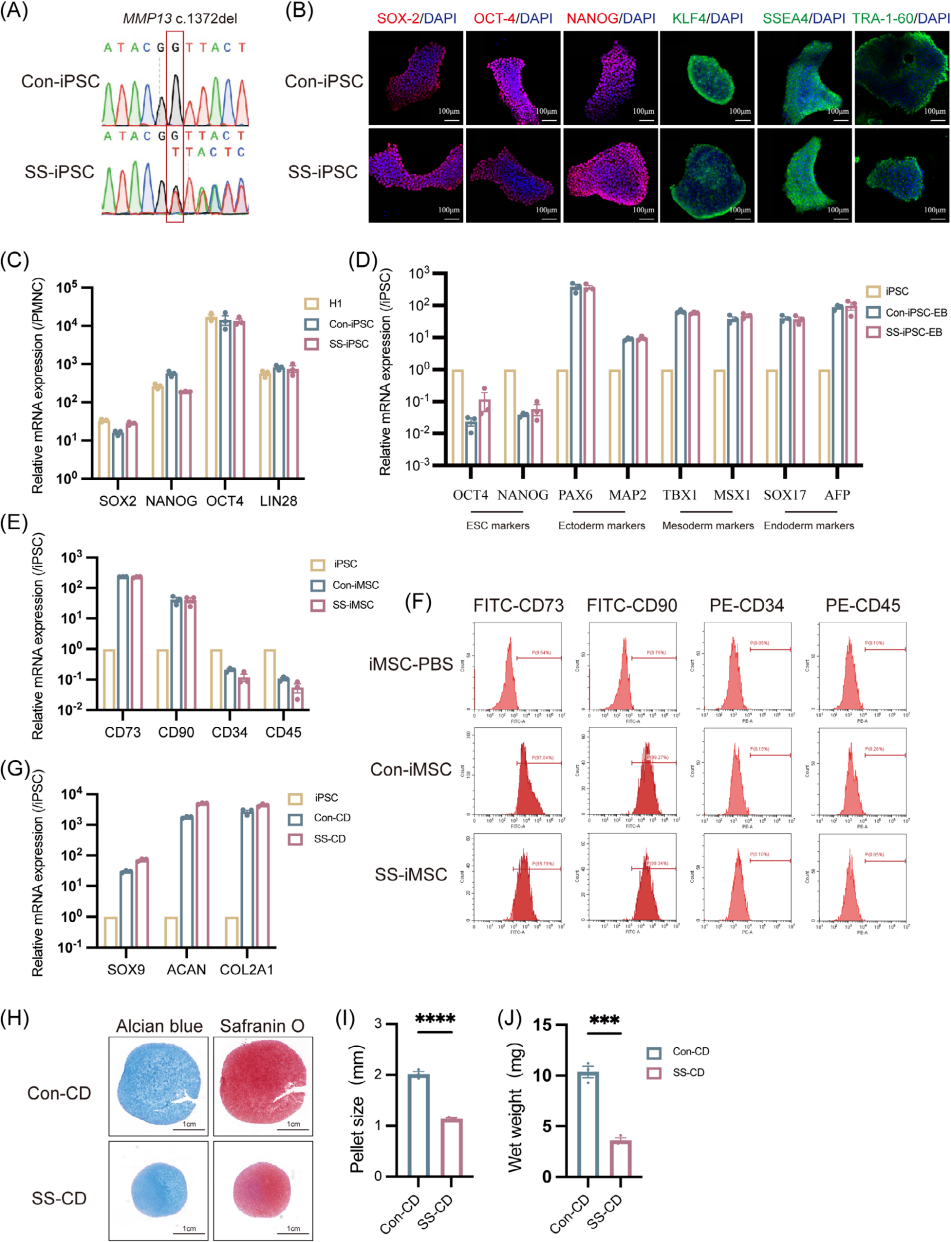
Identification of induced pluripotent stem cells (iPSCs) and differentiation of chondrogenic pellets. (A) Identification of iPSCs from peripheral blood mononuclear cells (PBMCs) of both control and patient. MMP13 variant is present in SS‐iPSCs but absent in Con‐iPSCs. (B) Representative immunostaining for SOX‐2, OCT‐4, NANOG, KLF4, SSEA4 and TRA‐1‐60. Nuclei are stained with 4′,6‐diamidino‐2‐phenylindole (DAPI) (scale bar, 100 µm). (C) The expression of iPSCs‐specific markers. These iPSCs highly expressed SOX2, NANOG, OCT4 and LIN28 (*n* = 3). The polymorphonuclear cells (PMNCs) served as a control cell. (D) Potential to differentiate into three germ layers. These iPSCs generated cellular derivatives expressing ectoderm markers (PAX6 and MAP2), mesoderm markers (TBX1 and MSX1) and endoderm markers (SOX17 and AFP). The expression of pluripotent markers (OCT4 and NANOG) was decreased (*n* = 3). iPSC served as a control. (E and F) Expression of mesenchymal stem cell (MSC)‐specific markers by RT‐PCR (*n* = 3) and flow cytometry. These MSCs highly expressed CD73 and CD90, while showing decreased expression of CD34 and CD45. (G) Identification of chondrogenic pellets. The expression of chondrogenic‐specific marker gene. These chondrogenic highly expressed SOX9, ACAN and COL2A1 (*n* = 3). (H) Histochemical staining of iPSC‐derived chondrocyte spheroids. Both Alcian blue (left) and Safranin O (right) staining showed positive, demonstrating the presence of glycosaminoglycans in the ECM of chondrocyte spheroids (scale bar, 1 cm). (I and J) Diameter and wet weight of chondrocyte spheroids derived from control‐ and patient‐derived MSCs (*n* = 3). Data are represented as mean ± standard error (SEM). ^*^ *p* < .05, ^**^ *p* < .01, ^***^ *p* < .001.

Peripheral blood mononuclear cells (PBMCs) were collected from the proband (II‐1) and a healthy family control (I‐2) within the SS pedigree. Sanger sequencing confirmed the presence of *MMP13* c.1372del variant in the patient sample, whereas this variant was absent in the control (Figure 2A). PBMCs were subsequently reprogrammed into iPSCs via nucleofection with episomal plasmids. The SS‐iPSCs and Con‐iPSCs were differentiated into chondrogenic via MSCs.^28^ Sequencing revealed the presence of *MMP13* variant in SS‐iPSCs but not in Con‐iPSCs. IF staining demonstrated that both cell lines were positive for pluripotency markers SOX‐2, OCT‐4, NANOG, KLF4, SSEA4 and TRA‐1‐60 (Figure 2B). Analysis by qPCR revealed comparable high expression of marker genes in both iPSC groups, indicating that the *MMP13* variant did not affect pluripotency gene expression (Figure 2C). Karyotyping also confirmed a normal female chromosomal complement (46, XX) (Figure S1A). Alkaline phosphatase positivity (Figure S1B), along with the ability to differentiate into ectodermal, mesodermal and endodermal lineages in embryoid body assay (Figure 2D), demonstrated that high‐quality iPSCs had been successfully generated.

Subsequently, flow cytometry confirmed that the generated MSCs expressed CD73 and CD90 but not CD34 or CD45 (Figure 2F), consistent with their mesenchymal identity. Additionally, qPCR analysis showed comparable differentiation efficiency between two groups (Figure 2E), indicating that the *MMP13* variant did not affect MSC differentiation potential. Then, chondrogenic pellets were identified by qPCR, Alcian blue staining and safranin staining. qPCR analysis revealed high expression of marker genes, including SOX9, ACAN and COL2A1, without significant differences between two chondrogenic pellets groups (Figure 2G). Histological examination of chondrogenic pellets, in both mutant and control groups, revealed similar safranin O and Alcian blue staining patterns (Figure 2H). These results indicated that the chondrogenic pellet model was successfully generated for further investigation. Chondrocyte pellets differentiated for 42 days were harvested for diameter measurement and wet weight analysis. Compared with the control, chondrocyte pellets from the patient had markedly reduced diameters (Figure 2I) and wet weights (Figure 2J). Consistent results were obtained by differentiating distinct iPSC clones derived from the same individual, which thereby excluded the potential confounding effects arising from the iPSCs’ intrinsic characteristics.

### 3.4 Generation of Mmp13^R459fs^ variant mice via CRISPR/Cas9

**FIGURE 3:**
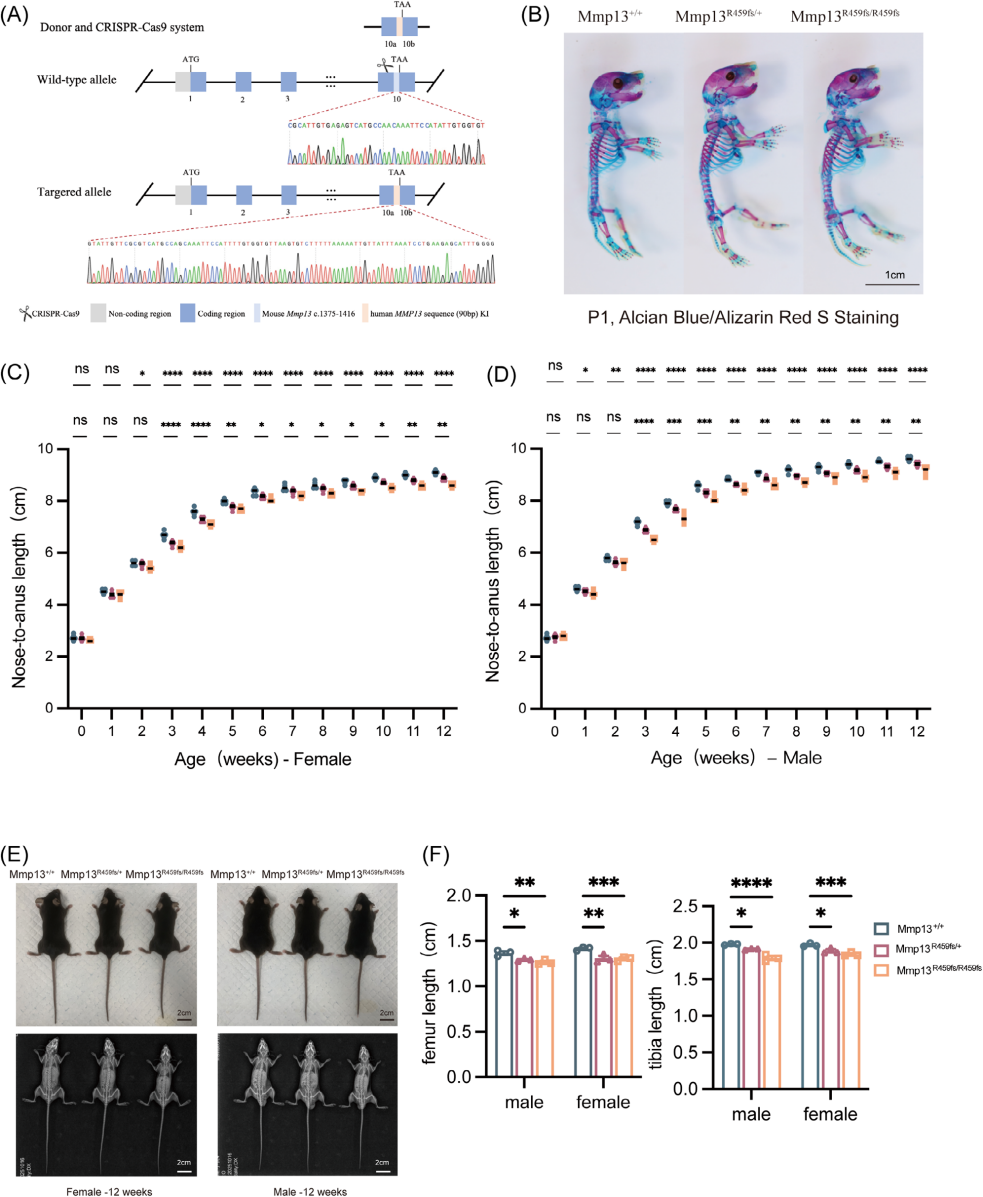
CRISPR/Cas9‐mediated mutagenesis to introduce the R459fs variant in MMP13 in mice. (A) Schematic diagram of CRISPR/Cas9‐mediated generation of mice with homologous variants. The murine Mmp13 sequence (c.1375‐1416) was knocked out, and a 90 bp human MMP13 sequence was knocked in. (B) Alcian blue/Alizarin Red S staining of mice with Mmp13^+/+^, MMP13^R459fs/+^ and Mmp13^R459fs/R459fs^ at postnatal day 1 (P1) (scale bar, 1 cm). (C and D) Anogenital length growth curves of three genotypes (wild‐type, Mmp13 heterozygous, Mmp13 homozygous) from P1 to 12 weeks of age. Mmp13^R459fs/R459fs^ mice exhibit poor length gain after 2 weeks. *n* = 5 for each group at each time point. Left: females; right: males. Source data are provided as a source data file. (E) Photographs and X‐ray images of 12‐week‐old mice with three genotypes. Left: female mice; right: male mice (scale bar, 2 cm). (F) Quantification of femur and tibia lengths in 12‐week‐old mice with three genotypes, measured in X‐ray images (*n* = 3). Data are presented as mean ± standard error (SEM). ^*^ *p* < .05, ^**^ *p* < .01, ^***^ *p* < .001.

To determine the effect of *MMP13* gene variant on the skeletal development, we established a precision‐edited mouse model (Mmp13^R459fs^) carrying the homologous variant, corresponding to human MMP13^R458fs^ variant. Human *MMP13* c.1372del results in an 18‐amino‐acid extension of the encoded amino acid sequence, whereas orthologous mouse *Mmp13* c.1375del variant led to premature termination of the amino acid sequence. To ensure that the mice carried the homologous variant identified in the proband, *Mmp13* c.1375‐1416 region was knocked out, and human *MMP13* c.1372‐1461 was knocked in via CRISPR/Cas9 (Figure 3A).

### 3.5 R459fs variant in *Mmp13* results in shortening of body length in mice

Mutant mice were viable and exhibited no overt developmental defects at birth. As shown by Alcian blue/alizarin red S staining, at postnatal day 1 (P1), Mmp13^R459fs/+^ and Mmp13^R459fs/R459fs^ mice had no significant abnormalities in the skull, scapula, humerus, forelimbs and hindlimbs as compared to the WT littermates (Figure 3B). The nose‐to‐anus length was measured in both male and female mice from all three groups and compared with sex‐matched WT littermates (Figure 3C,D). Beginning at 2 weeks of age, Mmp13^R459fs/+^ mice showed significantly reduced nose‐to‐anus length and smaller stature relative to sex‐matched WT littermates, whereas no significant differences were observed at birth and at 1 week of age. This growth deficit peaked at 3 weeks of age, when heterozygous mutant mice showed the most pronounced reduction in nose‐to‐anus length. After 6 weeks of age, the dwarfism phenotype was improved significantly, but their final nose‐to‐anus length remained smaller than that of WT littermates. Mmp13^R459fs/R459fs^ mice exhibited a shorter body length earlier, and the reduction in body length was more evident than that in Mmp13^R459fs/+^ mice. Then, both males and females of 12‐week‐old mice received measurement of lengths of the femurs and tibias in each group (Figure 3E). Results showed that the femurs and tibias of Mmp13^R459fs/+^ and Mmp13^R459fs/R459fs^ mice were significantly shorter than those of WT littermate, with the reduction being more evident in Mmp13^R459fs/R459fs^ mice (Figure 3F). Overall, these data suggested that the expression of R459fs variant resulted in reduced body length in mice.

### 3.6 Growth plate abnormalities in *Mmp13* ^R459fs^ variant mice

**FIGURE 4:**
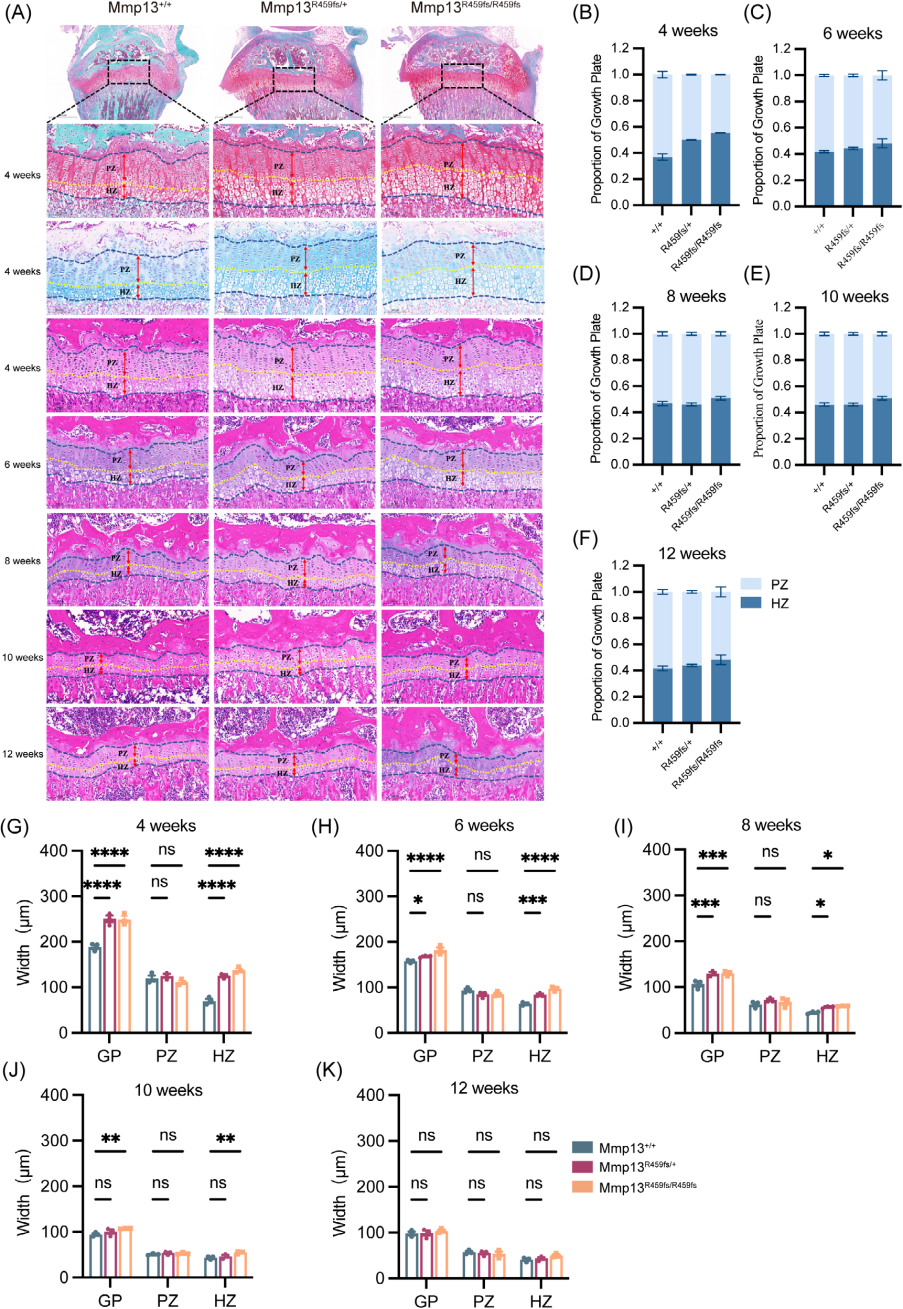
The R459fs variant in Mmp13 results in growth plate abnormalities in female mice. (A) Representative histological images of growth plates from Mmp13^+/+^, Mmp13^R459fs/+^ and Mmp13^R459fs/R459fs^ mice at 4, 6, 8, 10 and 12 weeks of age (stained with Safranin O/Fast Green, Alcian blue and haematoxylin‒eosin [HE] staining). Dashed boxes indicate the magnified regions, with red lines denoting the thickness of proliferative zone (PZ) and hypertrophic zone (HZ) in the growth plate (GP) (scale bar, 100 µm). (B‒F) Proportion of PZ and HZ in the growth plate of mice at 4, 6, 8, 10 and 12 weeks of age. (G‒K) Quantification of width (µm) of GP, PZ and HZ in *Mmp13* mutant mice at 4, 6, 8, 10 and 12 weeks (*n* = 3). Data are represented as mean ± standard error (SEM). ^*^ *p* < .05, ^**^ *p* < .01, ^***^ *p* < .001. This figure depicts growth plate staining of female mice, with the corresponding staining of male mice in the supporting figures.

To assess growth plate morphology, hindlimbs from *Mmp13* variant mice and WT littermates were collected at multiple time points between 4 and 12 weeks of age. Serial sections were then subjected to Safranin O/Fast Green, Alcian blue and HE staining for histological evaluation (Figure 4A‐F). Growth plate length was significantly increased in both Mmp13^R459fs/+^ and Mmp13^R459fs/R459fs^ mice as compared to WT littermates in hindlimb long bone sections, with the most pronounced difference observed at 4 weeks of age. In Mmp13^R459fs/R459fs^ mice, the proximal tibial growth plate was significantly elongated as compared to WT littermates, with overall length increasing by 31% and the hypertrophic zone expanding by 97%. In contrast, the proliferative zone was comparable in length across three genotypes. These abnormalities resolved with age: no significant differences were observed between Mmp13^R459fs/R459fs^ and WT littermates by 10 weeks of age, and by 12 weeks, and the total growth plate, proliferative zone and hypertrophic zone lengths were comparable across all three genotypes (Figure 4G‒K). However, the femurs of Mmp13^R459fs/+^ and Mmp13^R459fs/R459fs^ mice were also shorter than those of WT littermates at 12 weeks of age. Same results were observed in the length of tibias. The abnormal phenotype of growth plate was basically similar between male and female mice. Inada et al. reported that Mmp13^−/−^ mice exhibited disorganised and poorly aligned chondrocyte columns relative to WT littermates.^17^ In contrast, mice harbouring Mmp13 ^R459fs^ variant showed columnar alignment of chondrocytes. These phenotypes are consistent across male mice (Figure S2).

### 3.7 MMP13 variant impairs protein secretion

**FIGURE 5:**
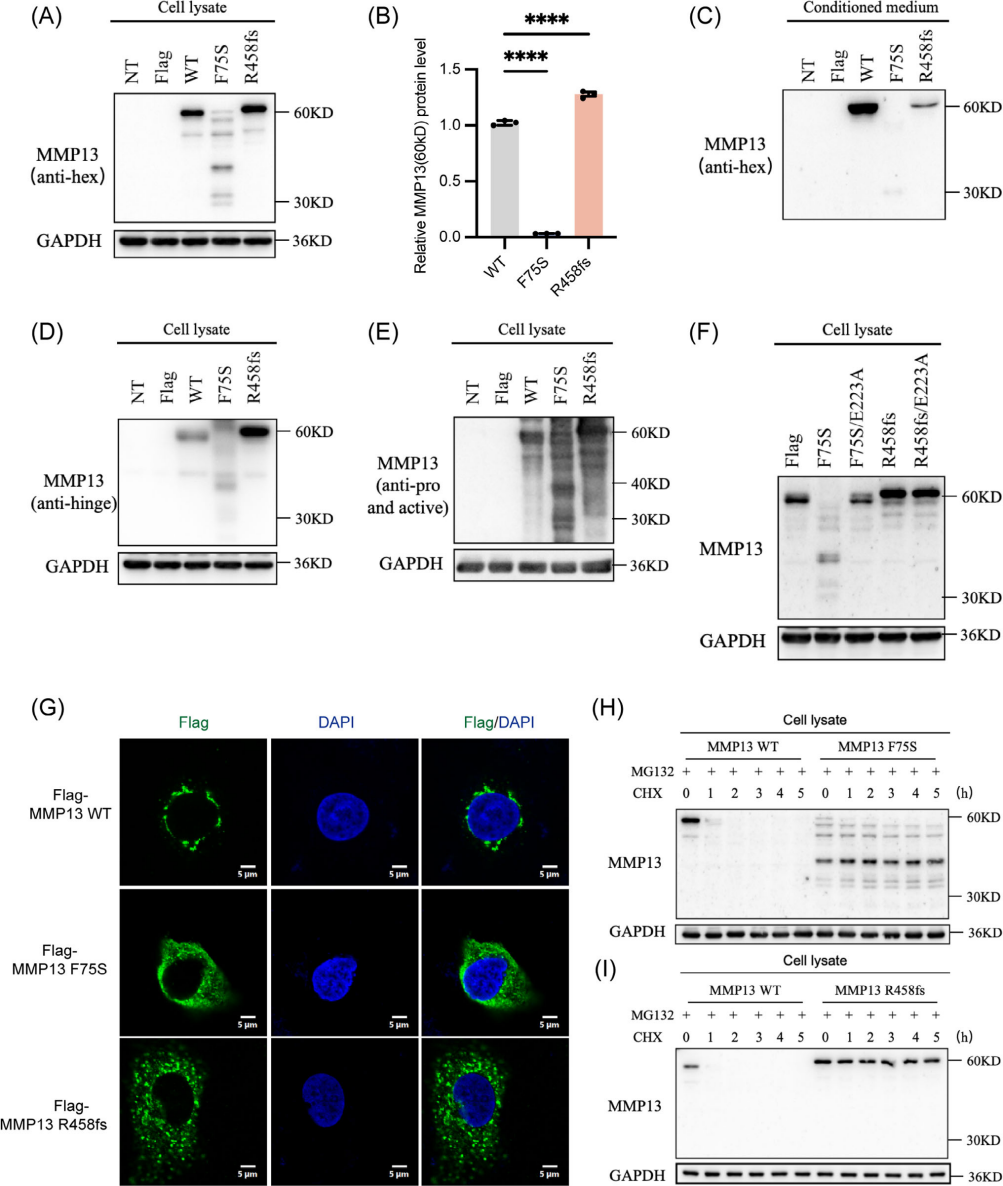
MMP13 R458fs variant leads to its intracellular accumulation and poor secretion. (A‒C) Impaired processing, intracellular accumulation and poor secretion of MMP13 R458fs variant in HEK293T cells. Western blotting of MMP13 protein abundance in the cell lysates and conditioned medium of HEK293T cells transfected with indicated plasmids after 48 h. Full‐length MMP13 WT and MMP13 R458fs were secreted into conditioned medium, whereas only fragments of MMP13 F75S with low molecular mass were secreted. Anti‐hex domain antibody was used (*n* = 3). (D) As in A, but anti‐hinge domain antibody was used. (E) As in A, but anti‐pro domain and active antibody was used. (F) Effects of E223A variant. F75S/E223A MMP13 was not autoactivated and autodegradated. (G) Immunofluorescence staining using the anti‐FLAG showed accumulated R458fs MMP13 inside the transfected HEK293T cells. The nuclei were stained by DAPI (blue) (scale bar, 5 µm). (H and I) Cumulative MMP13 protein after cycloheximide (CHX) and MG132 treatment. After CHX and MG132 treatment for 5 h, the F75S and R458fs MMP13 proteins intracellularly accumulated. Data are represented as mean ± standard error (SEM). ^*^ *p* < .05, ^**^ *p* < .01, ^***^ *p* < .001.

To evaluate whether MMP13 R458fs variant exhibited intracellular autoactivation, autodegradation and impaired MMP13 protein secretion, Flag‐tagged WT and mutant (p.F75S and p. R458fs) human MMP13 were overexpressed in cell lines via plasmid‐based delivery. After 48‐h transfection in HEK293T cells, conditioned media and cells were collected separately. For MMP13 R458fs variant cells, a lower amount of MMP13 protein was found in the conditioned media (Figure 5C), while increased intracellular protein retention was noted in cells transfected (Figure 5A,B), and the MMP13 protein was not degraded intracellularly on WB and IF staining (Figure 5G). Nevertheless, degraded misfolded protein fragments of MMP13 F75S mutant (approximately 30 kDa) were secreted. The band corresponding to the active MMP13 enzyme (40 kDa) was detected using an anti‐active MMP13 antibody (18165‐1‐AP, Proteintech). Consistent results were obtained with three distinct anti‐MMP13 antibodies (Figure 5D,E). Within the zinc‐binding motif (HEXXGH) of metalloproteinases, the conserved glutamic acid residue (E) is essential for its catalytic activity; substitution of this residue with alanine (E223A) has been shown to render other MMPs enzymatically inactive. Consistent with this, MMP13 F75S/E223A double variant was expressed at levels comparable to those of MMP13 WT and secreted normally. Notably, the introduction of E223A variation abolished the intracellular autoactivation and autodegradation phenotypes observed with F75S variant alone. However, MMP13 R458fs/E223A was expressed at a level similar to MMP13 R458fs. Thus, we speculated that MMP13 R458fs variant did not activate the catalytically active residue E223 (Figure 5F).

To examine whether MMP13 variants exhibit secretory defects, cells overexpressing MMP13 WT and MMP13 variants (F75S and R458fs) were treated with cycloheximide (100 µg/mL), a protein synthesis inhibitor, along with the proteasome inhibitor MG132 (10 µM). The MMP13 F75S and R458fs variants showed prolonged intracellular retention relative to the MMP13 WT cells (Figure 5H,I). These findings suggested that impaired protein secretion was the potential cause of reduced MMP13 level. This secretory defect likely accounts for both reduced extracellular MMP13 and its intracellular accumulation. The proband's clinical phenotype may be associated with the functional domain affected by the variant. In this family, individuals with *MMP13* variant exhibited no significant skeletal abnormalities in childhood beyond SS. Compared with previously reported cases with *MMP13* variants, their clinical phenotype was milder, which may be attributable to the variant site.

### 3.8 MMP13 variant activates ER stress by interacting with HSPA5

**FIGURE 6:**
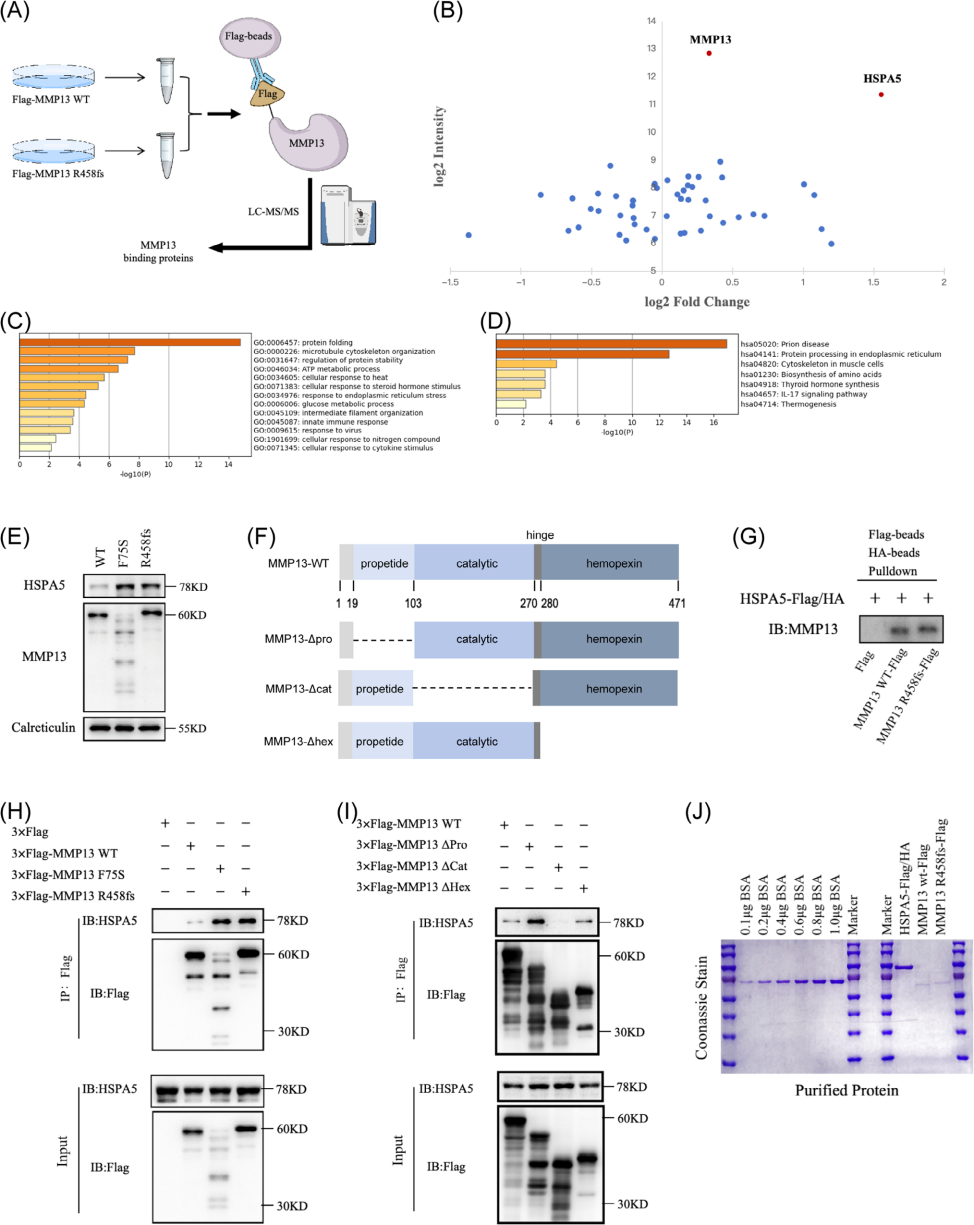
Co‐immunoprecipitation coupled with mass spectrometry (Co‐IP/MS) results and identification. (A) Schematic illustration of the Co‐IP combined LC‒MS/MS for MMP13 substrate screening. HEK293T cells were transfected with Flag‐MMP13 wild‐type (WT) or Flag‐MMP13 R458fs plasmids for 48 h. Flag beads were added to the cell samples for Co‐IP. The binding proteins were extracted, digested to peptides, and then subjected to LC‒MS/MS. (B) 2D plot highlights the differentially abundant proteins identified by MS in MMP13 R458fs as compared to MMP13 WT. Fifty proteins with the highest expression were shown in the plot. (C and D) Gene Ontology (GO) and kyoto encyclopedia of genes and genomes (KEGG) pathway enrichment analysis of top 50 proteins with the most significant interaction with MMP13 identified by Co‐IP/MS. (E) Expression of HSPA5 and MMP13 in endoplasmic reticulum (ER) fractions on Western blotting. HEK293T cells were transfected with MMP13 WT, MMP13 F75S or MMP13 R458fs plasmids for 48 h, and then harvested for ER isolation. Calreticulin was used as a reference gene for normalisation. (F) Schematic representation of MMP13 truncations. The deletions of propetide, catalytic or haemopexin domain of MMP13 were inserted into the corresponding p3×Flag plasmids (MMP13‐Δpro, MMP13‐Δcat and MMP13‐Δhex, respectively). (G) Interaction between MMP13 WT, or MMP13 R458fs and HSPA5 was determined in vitro. Purified MMP13 WT‐Flag, or MMP13 R458fs‐Flag and HSPA5‐Flag/HA were subjected to sequential immunoprecipitation: first with Flag beads, followed by HA beads the next day. The samples were then washed in washing buffer containing 300 mM NaCl before Western blotting. (H) The interaction between MMP13 WT, MMP13 F75S or MMP13 R458fs and HSPA5 was detected by exogenous Co‐IP. MMP13 WT, MMP13 F75S or MMP13 R458fs were co‐expressed in HEK293T cells. After 48 h, cells were collected using a cytobrush. The lysates were then precipitated with Flag beads, and Co‐IP complexes were analysed using HSPA5 and Flag antibodies. (I) Interaction between MMP13 truncations and HSPA5 was determined by Co‐IP. MMP13 WT and each of the three truncations of MMP13 (MMP13‐Δpro, MMP13‐Δcat and MMP13‐Δhex) were co‐expressed in HEK293T cells. As in H. (J) Coomassie staining of eukaryotic purified HSPA5‐Flag/HA, MMP13 WT‐Flag and MMP13 R458fs‐Flag.

The mechanism by which intracellular aggregation of MMP13, coupled with reduced secretion of catalytically active MMP13, ultimately contributes to SS remains unclear. To clarify the underlying mechanisms, Co‐IP/MS was performed to identify the most differentially expressed protein between WT and mutant groups (Figure 6A). Results showed HSPA5 was significantly overexpressed in mutant cells (Figure 6B).

HSPA5, also known as 78‐kDa glucose‐regulated protein (GRP78) or binding immunoglobulin protein (BiP), is a member of heat shock protein 70 (HSP70) family. In contrast to cytosolic HSPs, GRP78 mostly resides in the ER, where it participates in protein trafficking, folding and ER‐associated degradation (ERAD).^29^ Subsequent pathway analysis of enriched proteins through Gene Ontology and kyoto encyclopedia of genes and genomes (KEGG) identified substantial correlations with biological processes such as protein folding and ER stress response (Figure 6C,D).

The majority of proteins encoded by genes associated with SS were components of ECM or regulators of ECM metabolism.^30^ Pathogenic variants in these genes may impair skeletal development by causing abnormal ECM protein interactions, diminishing protein secretion, or inducing ER stress from misfolded proteins, thereby undermining the structural integrity of ECM and disrupting the biological signalling essential for skeletal growth and homeostasis.^31^ Nevertheless, the pathogenic mechanism associated with MMP13‐related SS remains unexplained. Our findings from Co‐IP/MS suggested a novel pathogenic hypothesis: pathogenic variants of *MMP13* gene induce SS via increasing unfolded proteins, upregulating HSPA5, elevating ER stress and activating unfolded protein response (UPR).^32^

Transfection was performed in HEK293T cells to determine whether the decreased secretion of mutant MMP13 results from increased ER retention, as seen in other mutant ECM proteins.^33^ After 48‐h transfection, cells were harvested, and ER fractions were extracted for WB. Significantly increased HSPA5 was found in the ER of both MMP13 F75S and R458fs mutant cells relative to WT controls. Simultaneously, MMP13 R458fs cells showed increased ER retention, whereas the MMP13 F75S mutant cells displayed intracellular degradation (Figure 6E). Co‐IP indicated a substantially enhanced interaction between MMP13 mutant cells and HSPA5 cells as compared to MMP13 WT cells (Figure 6H). Moreover, total HSPA5 expression was upregulated in all cells producing MMP13 mutants. To figure out the particular domain facilitating MMP13‒HSPA5 interactions, a variety of MMP13 truncation mutants were constructed (Figure 6F). Co‐IP revealed direct interaction between MMP13 catalytic domain and HSPA5 (Figure 6I). To determine whether MMP13 directly interacts with HSPA5, Flag‐tagged MMP13 and Flag‐HA‐tagged HSPA5 were expressed and purified using a eukaryotic system. These purified proteins were then subjected to sequential two‐step pull‐down assay: first incubation with Flag beads, followed with HA beads the next day. Results confirmed a direct interaction between MMP13 and HSPA5, as evidenced by strong in vitro binding (Figure 6G,J).

Accumulation of misfolded or unfolded proteins within ER may trigger ER stress,^34^, ^35^ which in turn activates an adaptive cellular response known as UPR.^36^ UPR orchestrates broad modifications to the secretory pathway, including regulation of protein synthesis, translocation into ER, folding, maturation, quality control, trafficking and degradation of misfolded proteins via autophagy and ERAD. While selective activation of UPR may be cytoprotective, persistent UPR activation due to prolonged ER stress may lead to adverse consequences, including cellular apoptosis.^37^, ^38^, ^39^

The mutation aggravated ER stress in chondrocytes differentiating from iPSCs and in growth plate cells of mice in vivo.

**FIGURE 7:**
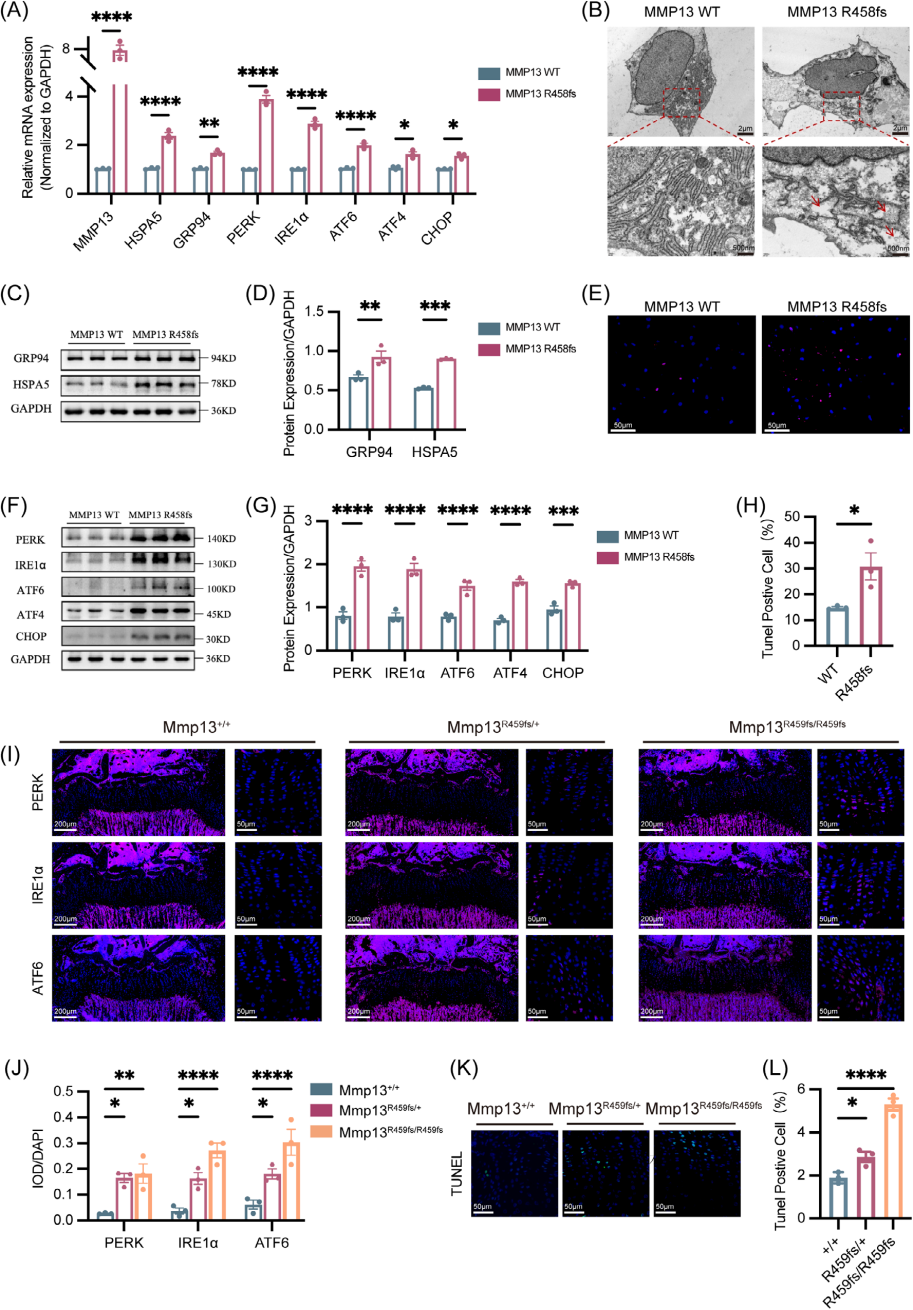
Mutation led to endoplasmic reticulum (ER) stress aggravation in Chondrocytes differentiating from induced pluripotent stem cells (iPSCs) and in the growth plate cells of mice in vivo. (A) mRNA levels of MMP13, HSPA5, GRP94, PERK, IRE1α, ATF6, ATF4 and CHOP in control‐ and patient‐derived chondrocytes (*n* = 3). (B) Representative transmission electron microscopy (TEM) images of chondrocyte pellets (scale bar for upper images, 2 µm; scale bar for enlarged images, 500 nm). (C) Western blotting of GRP94 and HSPA5 (*n* = 3). (D) Quantified analysis of protein bands of GRP94 and HSPA5. (E) TUNEL staining showed more apoptotic cells in MMP13 R458fs mutant chondrocytes than in control chondrocytes (scale bar, 50 µm). (F) Western blotting analysis of PERK, IRE1α, ATF6, ATF4 and CHOP. (G) Quantification of protein bands of PERK, IRE1α, ATF6, ATF4 and CHOPb (*n* = 3). (H) Quantification of TUNEL‐positive cells in paraffin sections from control‐ and patient‐derived chondrocytes (*n* = 3). (I) Immunofluorescence analysis of PERK, IRE1α and ATF6 expression in the growth plates from Mmp13^+/+^, Mmp13^R459fs/+^ and Mmp13^R459fs/R459fs^ mice at 4 weeks of age (scale bar, 200 and 10 µm). (J) Quantification of the integrated optical density (IOD)/DAPI stain intensity levels of PERK, IRE1α and ATF6 in the growth plates of mice (*n* = 3). (K) TUNEL staining of the lower limbs of the Mmp13^+/+^, Mmp13^R459fs/+^ and Mmp13^R459fs/R459fs^ mice at 4 weeks of age (scale bar, 50 µm). (L) Quantification of the percentage of TUNEL‐positive cells in the growth plate shown in panel (J). Data are presented as mean ± standard error (SEM). ^*^ *p* < .05, ^**^ *p* < .01, ^***^ *p* < .001.

Our previous Co‐IP/MS findings revealed significantly elevated HSPA5 level in cells expressing MMP13 variant compared with controls, leading us to hypothesise that *MMP13* variants drive SS via HSPA5‐mediated ER stress. To test this hypothesis, we examined whether intracellular retention of mutant protein induced ER stress and triggered abnormal cell death in iPSC‐derived chondrogenic cells. ER stress and UPR markers were detected at mRNA (Figure 7A) and protein levels (Figure 7C,D,F,G). *MMP13* mutant cells showed increased expression of chaperones HSPA5 and GRP94, as well as increased expression of PERK, IRE1α, ATF6, ATF4 and CHOP. To investigate the impact of intracellular MMP13 accumulation on human chondrogenicity, transmission electron microscopy was performed to assess ER, and results showed that ERs were mildly to severely enlarged in the mutant group (Figure 7B). In addition, sustained ER stress induces chondrocyte apoptosis. Patient‐derived chondrocytes exhibited a marked increase in TUNEL‐positive cells relative to controls (Figure 7E,H). In growth plate cells of R459fs‐mutant mice, the expression of PERK, IRE1α and ATF6 was increased compared with WT mice (Figure 7I,J). Consistent with elevated ER stress‐induced cell apoptosis, a similar increase in growth plate cells was observed in mutant mice (Figure 7K,L).

## 4 DISCUSSION

In this study, genetic analysis was performed in an SS family, a frameshift variant in *MMP13* was identified as the genetic cause of an autosomal dominant inheritance of SS, and its pathogenicity was confirmed in patient‐derived iPSCs and a mouse model carrying the homozygous variant. The precision‐edited mice Mmp13^R459fs^ partially recapitulated the defective SS phenotypes observed in the pedigree subject. In Mmp13 ^R459fs^ mice, the skeletal alterations manifested only in the growth phase, similar to those in *Mmp13* knockout mice. However, based on morphological changes in the growth plate of Mmp13^R459fs^ mice, the growth plate phenotype improved earlier than in *Mmp13* knockout mice, where it did not improve until 12 weeks of age.^22^ *MMP13* gene variants cause SEMD_MO_ or Spahr type metaphyseal dysplasia. These conditions are defined by a constellation of skeletal abnormalities: metaphyseal lesions of moderate to severe degree, mild epiphyseal changes, pear‐shaped vertebrae in childhood, rhizomelic limb shortening and varus angulation of the legs. The skeletal phenotype is particularly prominent during periods of rapid childhood growth and tends to gradually improve in adolescence. However, affected individuals often exhibit a final adult height below their genetic potential.^40^ In contrast, the proband and her father, who carried MMP13 R458fs variant, exhibited only SS, with no other manifestations of skeletal dysplasia. We hypothesise that this may be due to the MMP13 R458fs variant failing to activate E223, thereby abrogating intracellular autodegradation of MMP13 protein. Although the variant impairs secretion, a small amount of MMP13 secreted into the ECM consists of full‐length fragments, which likely accounts for the milder clinical phenotype in affected individuals. In addition, although statistically significant differences were observed in the anogenital‒nasal length as well as the femoral and tibial lengths between mutant mice and WT mice in the mouse model, the magnitude of these differences was relatively modest. This may be associated with the domain of mutation site. Without additional comparable cases, it remains uncertain whether the above findings are coincidental, and their potential clinical relevance, therefore, cannot be firmly established.

A comprehensive understanding of the molecular mechanisms underlying MMP13‐related SS caused by *MMP13* variants may facilitate the development of novel therapeutic strategies for affected individuals. Our study, for the first time, reported that the MMP13 R458fs variant exhibited neither intracellular autoactivation nor autodegradation. Instead, cells expressing this mutant exhibited reduced MMP13 secretion and prominent intracellular retention of the protein. These findings indicate the variation disrupts normal protein processing and secretion. Given that MMP13 primarily functions as a secreted matricellular protein within the ECM, impaired secretion is likely the primary pathogenic mechanism underlying this disease.

Co‐IP/MS revealed HSPA5 as the most significantly enriched protein in cells expressing MMP13 variant as compared to controls. Accumulation of misfolded proteins in the ER triggers ER stress, activating UPR, a conserved signalling pathway that reconfigures transcriptional networks to restore proteostasis. Upon activation, UPR orchestrates a broad transcriptional program that modulates ER protein folding capacity to meet cellular demand. Thus, UPR represents a paradigm of homeostatic control mechanisms that dynamically adjust organellar function and abundance in response to physiological or developmental cues.^41^ The main outputs of UPR are the overexpression of ER resident chaperones, improved degradation and quality control of misfolded polypeptides, and reduction in global protein translation.^42^ Activation of UPR exerts comprehensive regulation over the secretory pathway, fundamentally modifying protein synthesis rates and ER translocation,^35^ influencing protein folding, maturation and quality control processes, reorganising protein trafficking dynamics, and facilitating the clearance of misfolded polypeptides through coordinating autophagy and ERAD mechanisms.^43^ When ER stress exceeds the proteostatic capacity of UPR, cells irrevocably activate apoptotic programs.

The specificity of SS resulting from *MMP13* variants is primarily attributed to the tissue‐specific expression and functional specialisation of this protein. MMP13 is mainly produced by chondrocytes and osteoblasts,^15^ and during embryonic development, its expression is restricted to skeletal structures, specifically the cartilaginous growth plate and the primary ossification centre.^17^ As a secreted ECM protein, MMP13 mediates collagen degradation and plays a critical role in bone formation and remodelling. Our study revealed that *MMP13* variants led to mutant protein retention in the ER. This retention, on one hand, induces ER stress specifically in chondrocytes, thereby impairing their function; on the other hand, it reduces the secretion of MMP13 into ECM, disrupting ECM remodelling. IF staining confirmed that pathological changes were predominantly localised to the growth plate—the essential site for longitudinal bone growth. Consequently, ER stress triggered by this variant disrupts key processes in skeletal development, contributing specifically to the SS phenotype. Collectively, the accumulation of mutant MMP13 protein in the ER triggers ER stress and maladaptive UPR signalling, resulting in harmful phenotypes in chondrocytes, which ultimately impairs endochondral ossification and long bone formation.^8^

In conclusion, *MMP13* c.1372del variant is classified as a pathogenic variant according to the ACMG/AMP classification criteria (Table S7), and this study extends the mutational spectrum and genotype/phenotype correlations of *MMP13* variants, indicating that patients may exhibit SS alone, without obvious imaging abnormalities in the vertebrae and epiphyses. Our study for the first time reveals that *MMP13* variants impair chondrocyte differentiation by triggering ER stress through inducing their retention in the ER. We constructed, for the first time, an iPSC model derived from patients with *MMP13* variant. This model provides a superior evaluation of rare patient variants, sheds light on genotype‒phenotype relationships, and uncovers potential pathogenic mechanisms associated with MMP13‐related SS, which are important for drug screening for different chondrodysplasia variants in the future.

## AUTHOR CONTRIBUTIONS

Huifei Lu, Xin Feng and Suping Dai conceptualised the study under supervision of Chunlin Wang and Qingfeng Yan. Chunlin Wang, Qingfeng Yan and Huifei Lu designed the study, drafted the manuscript and coordinated the project. Huifei Lu, Yilin Zhu and Yonghua Chen collected the clinical samples. Huifei Lu, Xin Feng and Suping Dai performed all experiments. Huifei Lu, Xin Feng and Ke Yuan performed the data analysis. Huifei Lu and Suping Dai wrote the manuscript. Chunlin Wang, Qingfeng Yan, Jianfang Zhu and Yanlan Fang contributed to the review and editing of the manuscript. All the authors read and approved the final version of the manuscript. The authorship order reflects their respective contributions.

## CONFLICT OF INTEREST STATEMENT

The authors declare no competing financial interests. Furthermore, they have no personal relationships that could have inappropriately influenced the work described in this study.

## ETHICS STATEMENT

All human samples were obtained and processed in accordance with the principles of the Declaration of Helsinki and the protocols approved by the Ethics Committee of The First Affiliated Hospital of Zhejiang University School of Medicine (no. 1468 in 2024). Written informed consent was provided by the proband's parents prior to their inclusion in the research. All animal experiments were conducted with approval from the Institutional Animal Care and Use Committee of The First Affiliated Hospital of Zhejiang University School of Medicine (no. 015 in 2026).

## Supporting information



Supporting information

## Data Availability

This manuscript and its Supporting Information contain all data necessary to support the conclusions of this study. All materials utilised in this research are either obtainable from commercial suppliers or can be requested from the corresponding authors under reasonable terms.
