# Supplementary material for: A novel MMP13 frameshift variant causes short stature via enhanced MMP13–HSPA5 interaction and activated endoplasmic reticulum stress
Source: Clin Transl Med. 2026 Mar 24;16(3):e70648. doi: 10.1002/ctm2.70648 (PMC13093721; doi:10.1002/ctm2.70648)
Supplement: Supplementary file 1 — Supporting information [file CTM2-16-e70648-s001.docx]

**SUPPLEMENTARY** **MATERIALS**

**Generation** **of** **iPSCs**

The iPSCs were generated by transfection with episomal plasmids including pCXLE-hOCT3/4 (Addgene, Massachusetts, USA, Cat. no. #27076), pCXLE-hSK (Addgene, Cat. no. #27078), and pCXLE-hUL (Addgene, Cat. no. #27080) via LONZA 4D (Maryland, USA). About 14 days after transfection, the clones were picked out. The iPSC clones were cultured in mTesR1 medium (Stem cell Technologies, Vancouver, Canada) and sub-cultured when reaching 70% confluence. The expression of the pluripotent genes was detected via immunofluorescence and Quantitative Real-Time PCR. Karyotyping, alkaline phosphatase staining, and three germ layers differentiation were performed as reported previously. Cells were harvested to detect the MMP13 c.1372del variant. The forward primer was: TGGTGGCATGCACCTGTAAT; and the reverse primer was:GACAGACCATGTGTCCCATT.

**Enrichment of ER**

The ER Enrichment kit (Invent Biotechnologies) was used to isolate ER in accordance with the manufacturer’s guidelines. In brief, 3.0 × 107 cells were harvested, frozen at -80°C for 10 min, and subsequently supplemented with 550 μL of buffer A. Cell suspension was thereafter transferred to a filter cartridge and subjected to centrifugation twice. Next, the whole supernatant was transferred to a fresh tube and subjected to centrifugation. Following centrifugation, the supernatant was transferred to a new tube and 40 μL of buffer B was added, followed by centrifugation again. The pellet resuspended with cold buffer A. 40 μL of buffer C was added followed by vortexing briefly. The supernatant was transferred into a fresh tube and 400 μL of buffer D was added. After centrifugation, the pellet was reconstituted in buffer WA-009.

**SUPPLEMRNTAL FIGURES**


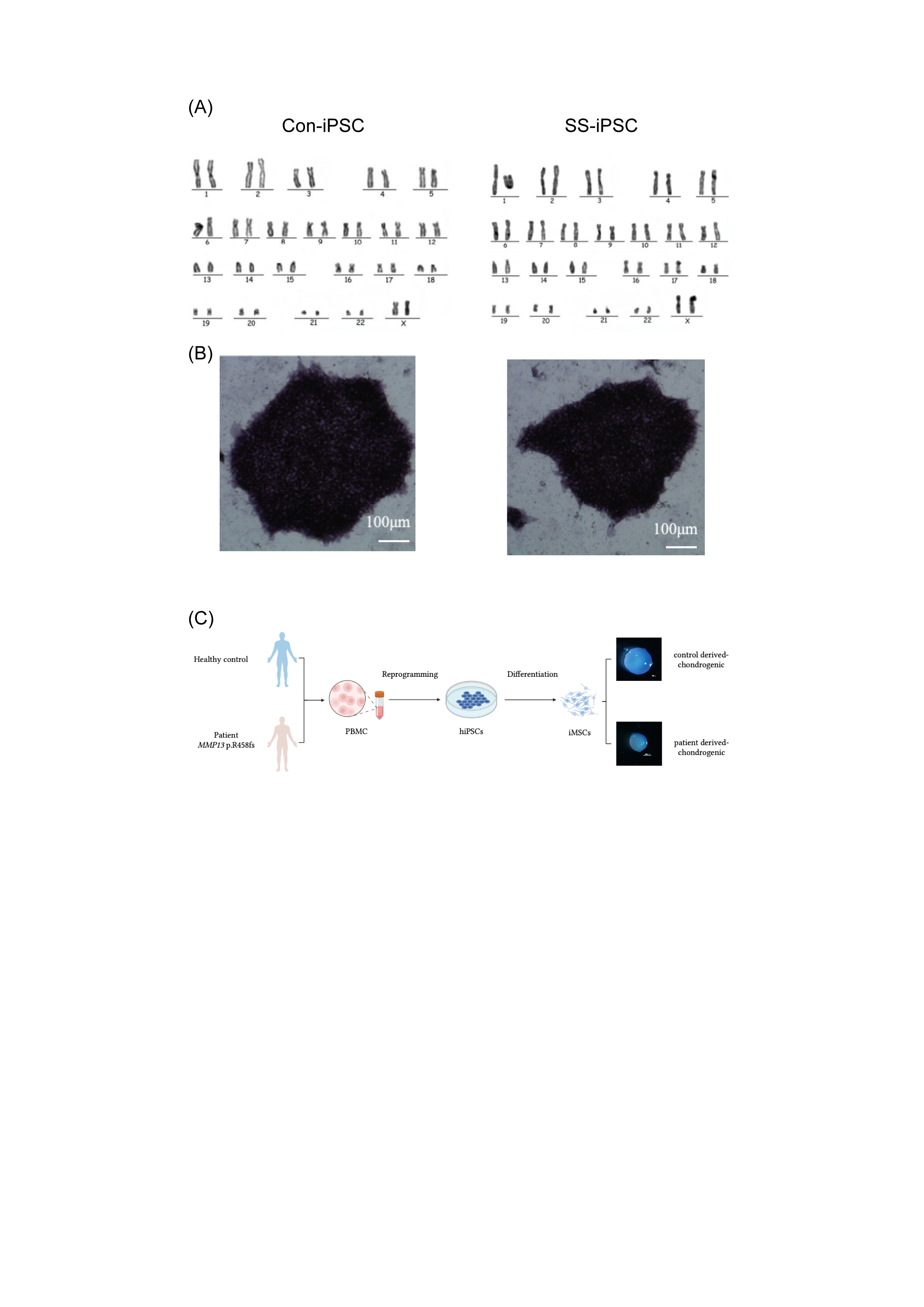


**Figure S1**

Generation and identification of iPSCs. (A) Karyotype analysis of iPSCs. These iPSCs had a normal karyotype (46, XX). (B) Alkaline phosphatase assay. These iPSCs were positively stained for alkaline phosphatase (scale bar, 100 µm). (C) Schematic representation of the chondrogenic differentiation of iPSCs.


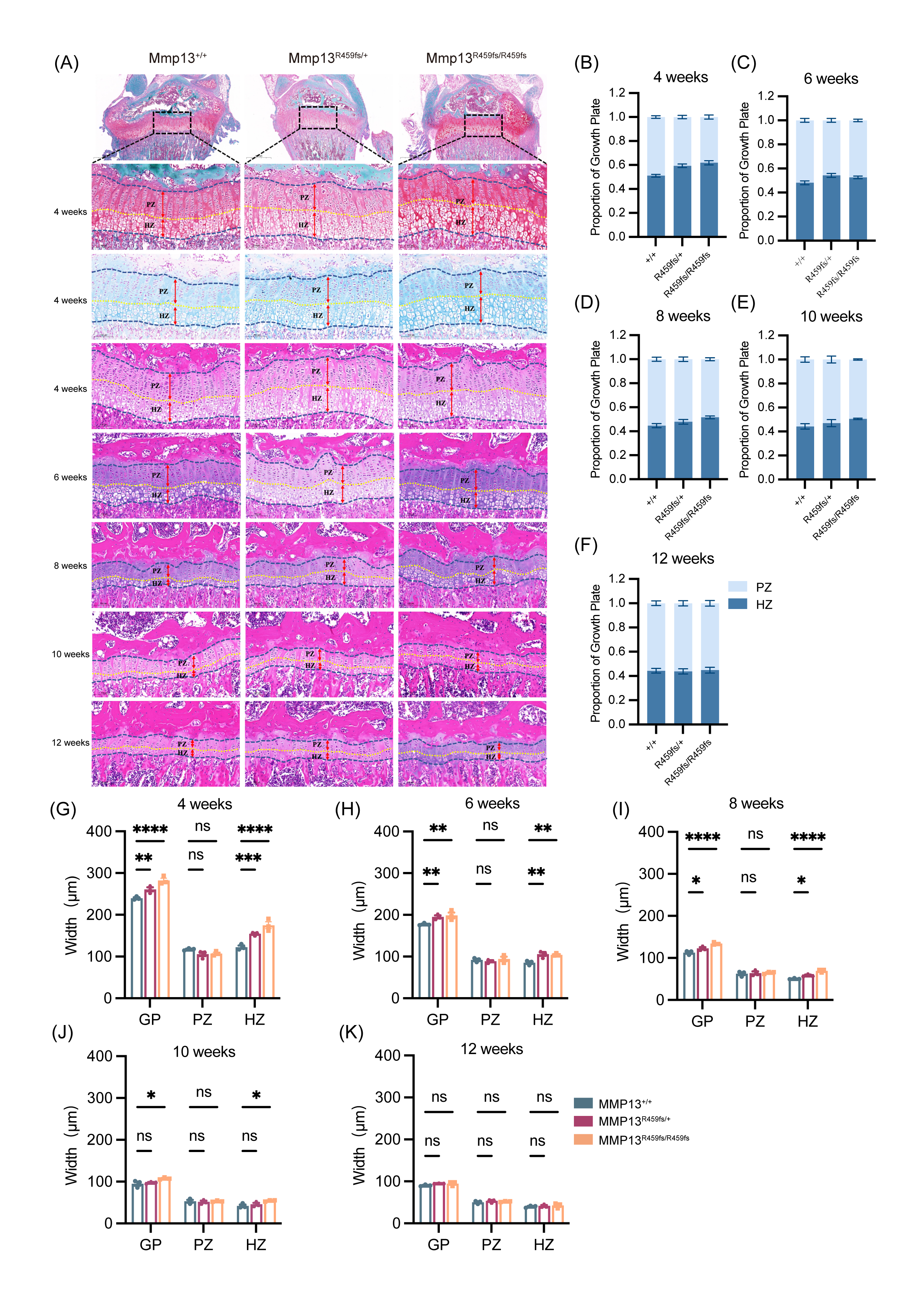


**Figure S2**

R459fs variant in *Mmp13* results in growth plate abnormalities in male mice. (A) Representative histological images of growth plates from Mmp13^+/+^, Mmp13^R459fs/+^, and Mmp13^R459fs/R459fs^ mice at 4, 6, 8, 10, and 12 weeks of age (stained with safranin O/fast green, alcian blue, and HE staining). Dashed boxes indicate the magnified regions, with red lines denoting the thickness of proliferative zone (PZ) and hypertrophic zone (HZ) in the growth plate (GP). (B-F) Proportion of PZ and HZ in the growth plate of mice at 4, 6, 8, 10, and 12 weeks of age (scale bar, 100 µm). (G-K) Quantification of width (μm) of GP, PZ, and HZ in *Mmp13* mutant mice at 4 weeks, 6 weeks, 8 weeks, 10 weeks, and 12 weeks (n=3). Data are presented as mean ± SEM. *P<0.05, **P<0.01, ***P<0.001.

**SUPLEMRNTAL TABLES**

Table S1. Three variants based on clinical assessment

| Gene | Variation | Type | Classification,  Review status |
| --- | --- | --- | --- |
| ARCN1 | NM_001655.5:c.16G>T(p.Ala6Ser) | Single nucleotide variant  (missense variant) | Benign |
| FGFR1 | NM_023110.3:c.1932C>T(p.Leu644=) | Single nucleotide variant  (synonymous variant) | Likely benign |
| MMP13 | NM_002427.4:c.1372del(p.Arg458fs) | Deletion  (frameshift variant) | Uncertain significance |

| **Items** | **Proband** | **Father** | **Mother** |
| --- | --- | --- | --- |
| Liver function | normal | | |
| renal function | normal | | |
| thyroid function | normal | | |
| calcium/phosphorus/ alkaline phosphatase | normal | | |
| IGF-1 | 182ng/ml | - | - |
| karyotype | 46，XX | 46，XY | 46，XX |
| bilateral lower limb radiographs | normal | | |
| anteroposterior pelvic radiographs | normal | | |

Table S2. Blood biochemical and X-ray

Table S3. The primers for MMP13 gDNA test.

|  |  | Sequence (5’→3’) |
| --- | --- | --- |
| MMP13  c.1372del | Forword | TGGTGGCATGCACCTGTAAT |
|  | Reverse | GACAGACCATGTGTCCCATT |

Table S4. Primers for vector construction.

|  |  | Sequence (5’→3’) |
| --- | --- | --- |
| *MMP13-*3×Flag | Forword | AATTAAGCTTGCGGCCGCGAATTCATCGATAatgcatccaggggtcctggct |
|  | Reverse | AGTCAGCCCGGGATCCTCTAGAGTCGACacaccacaaaatggaatttgctgg |
| *MMP13* F75S-3×Flag | Forword | gagaaatgcagtctttctCcggcttagaggtgactggcaa |
|  | Reverse | ttgccagtcacctctaagccgGagaaagactgcatttctc |
| *MMP13* E223A-3×Flag | Forword | tttcttgttgctgcgcatgCgttcggccactccttaggt |
|  | Reverse | acctaaggagtggccgaacGcatgcgcagcaacaagaaa |
| *MMP13* R459fs-3×Flag | Forword | AATTAAGCTTGCGGCCGCGAATTCATCGATAatgcatccaggggtcctggct |
|  | Reverse-1 | aacaccacaaaatggaatttgctggcatgacgcgaacaatacgttactccagatg |
|  | Reverse-2 | cttcaggatttaaataacaatttttaaaaagacacttaacaccacaaaatgga |
|  | Reverse-3 | TCAGCCCGGGATCCTCTAGAGTCGACccccaaatgctcttcaggatttaaat |
| *HSPA5-*3×HA | Forword | CGGTTCTATCGATTGAATTatgaagctctccctggtggcc |
|  | Reverse | GGGTAGTCGACTCTAGAGGATCCcaactcatctttttctgc |
| *MMP13* ΔPro-3×Flag | Forword | agctggactcattgtcgggcctacaatgttttccctcgaact |
|  | Reverse | agttcgagggaaaacattgtaggcccgacaatgagtccagct |
| *MMP13* ΔCat-3×Flag | Forword | ggttcctgatgtgggtgaagaagaccccaaccctaaacat |
|  | Reverse | atgtttagggttggggtcttcttcacccacatcaggaacc |
| *MMP13* ΔHex-3×Flag | Forword | AATTAAGCTTGCGGCCGCGAATTCATCGATAatgcatccaggggtcctggct |
|  | Reverse | TAGTCAGCCCGGGATCCTCTAGAGTCGACcgtttttggatgtttagggtt |

Table S5. Antibody used for western blot and IF.

| \| Antibody \|  \|  \| \| --- \| --- \| --- \| | Company | Lot |
| --- | --- | --- | --- | --- | --- |
| Anti-MMP13 | CST | #69926 |
| Anti-MMP13 | Protein tech | 18165-1-AP |
| Anti-MMP13 | Abcam | ab39012 |
| Anti-GAPDH | CST | #2118 |
| Anti-FLAG | CST | #14793 |
| Anti-HSPA5 | CST | #3177 |
| Anti-SOX-2 | CST | #3579 |
| Anti-OCT-4 | CST | #2750 |
| Anti-NANOG | CST | #4903 |
| Anti-KLF4 | Beyotime | C3252 |
| Anti-SSEA4 | CST | #4755 |
| Anti-TRA-1-60 | CST | #4746 |
| Anti-GRP94 | CST | #2104 |
| Anti-PERK | CST | #3292 |
| Anti-IRE1α | CST | #3294 |
| Anti-ATF6 | CST | #65880 |
| Anti-ATF4 | Protein tech | 10835-1-AP |
| Anti-CHOP | Protein tech | 15204-1-AP |

Table S6. Primers for Quantitative Real-Time PCR.

|  |  | Sequence (5’→3’) | |
| --- | --- | --- | --- |
| GAPDH | Forword | | GTCTCCTCTGACTTCAACAGCG |
|  | Reverse | | ACCACCCTGTTGCTGTAGCCAA |
| HSPA5 | Forword | | CTGTCCAGGCTGGTGTGCTCT |
|  | Reverse | | CTTGGTAGGCACCACTGTGTTC |
| GRP94 | Forword | | GGAGAGTCGTGAAGCAGTTGAG |
|  | Reverse | | CCACCAAAGCACACGGAGATTC |
| MMP13 | Forword | | CCTTGATGCCATTACCAGTCTCC |
|  | Reverse | | AAACAGCTCCGCATCAACCTGC |
| SOX2 | Forword | | AGGATAAGTACACGCTGCCC |
|  | Reverse | | TAACTGTCCATGCGCTGGTT |
| OCT4 | Forword | | GCCCGAAAGAGAAAGCGAAC |
|  | Reverse | | AACCACACTCGGACCACATC |
| NANOG | Forword | | TGAGATGCCTCACACGGAGA |
|  | Reverse | | GCAGAAGTGGGTTGTTTGCC |
| LIN28 | Forword | | CCAGTGGATGTCTTTGTGCACC |
|  | Reverse | | GTGACACGGATGGATTCCAGAC |
| PERK | Forword | | GTCCCAAGGCTTTGGAATCTGTC |
|  | Reverse | | CCTACCAAGACAGGAGTTCTGG |
| IRE1α | Forword | | CCGAACGTGATCCGCTACTTCT |
|  | Reverse | | CGCAAAGTCCTTCTGCTCCACA |
| ATF6 | Forword | | CAGACAGTACCAACGCTTATGCC |
|  | Reverse | | GCAGAACTCCAGGTGCTTGAAG |
| ATF4 | Forword | | TTCTCCAGCGACAAGGCTAAGG |
|  | Reverse | | CTCCAACATCCAATCTGTCCCG |
| CHOP | Forword | | GGTATGAGGACCTGCAAGAGGT |
|  | Reverse | | CTTGTGACCTCTGCTGGTTCTG |

Table S7. Reclassification of variants

| classification | Description |
| --- | --- |
| PVS1 | This variant is a frameshift variant, and the pathogenic mechanism of the *MMP13* gene variant is loss-of-function (LOF). |
| PS3 | Variants that have been confirmed to cause impaired gene function through in *vivo* and in *vitro* functional experiments. |
| PM2 | This variant was identified at low frequency in population databases. |
| PM4 | Base deletion results in changes in protein length. |
| PP1 | The variant cosegregates with the disease in the family. |
| PP4 | The phenotype of variant carriers is consistent with that of a monogenic inherited disease. |

In conclusion, the *MMP13* c.1372del variant is classified as a pathogenic variant according to the ACMG/AMP classification criteria.
